# Injective hydrogel loaded with liposomes-encapsulated MY-1 promotes wound healing and increases tensile strength by accelerating fibroblast migration via the PI3K/AKT-Rac1 signaling pathway

**DOI:** 10.1186/s12951-024-02666-3

**Published:** 2024-07-05

**Authors:** Chunhao Zhou, Zhihai Cai, Jialiang Guo, Chengfu Li, Chenghe Qin, Juanwen Yan, Dehong Yang

**Affiliations:** 1 Department of Orthopedics - Spinal Surgery, Nanfang Hospital, Southern Medical University, 1838 Guangzhou North Avenue, Guangzhou, 510515 China; 2 Department of Orthopedics - Traumatology, Nanfang Hospital, Southern Medical University, Guangzhou, 510515 China; 3 Department of Stomatology, Nanfang Hospital, Southern Medical University, Guangzhou, 510515 China

**Keywords:** Wound healing, Liposome, GelMA, PI3K/AKT, Rac1, Cell migration, hPTH(3–34)(29–34)

## Abstract

Failed skin wound healing, through delayed wound healing or wound dehiscence, is a global public health issue that imposes significant burdens on individuals and society. Although the application of growth factor is an effective method to improve the pace and quality of wound healing, the clinically approved factors are limited. Parathyroid hormone (PTH) demonstrates promising results in wound healing by promoting collagen deposition and cell migration, but its application is limited by potentially inhibitory effects when administered continuously and locally. Through partially replacing and repeating the amino acid domains of PTH(1–34), we previously designed a novel PTH analog, PTH(3–34)(29–34) or MY-1, and found that it avoided the inhibitory effects of PTH while retaining its positive functions. To evaluate its role in wound healing, MY-1 was encapsulated in liposomes and incorporated into the methacryloyl gelatin (GelMA) hydrogel, through which an injectable nanocomposite hydrogel (GelMA–MY@Lipo, or GML) was developed. In vitro studies revealed that the GML had similar properties in terms of the appearance, microstructure, functional groups, swelling, and degradation capacities as the GelMA hydrogel. In vitro drug release testing showed a relatively more sustainable release of MY-1, which was still detectable in vivo 9 days post-application. When the GML was topically applied to the wound areas of rat models, wound closure as well as tensile strength were improved. Further studies showed that the effects of GML on wound repair and tensile strength were closely related to the promotion of fibroblast migration to the wound area through the controlled release of MY-1. Mechanically, MY-1 enhanced fibroblast migration by activating PI3K/AKT signaling and its downstream molecule, Rac1, by which it increased fibroblast aggregation in the early stage and resulting in denser collagen deposition at a later time. Overall, these findings demonstrated that the nanocomposite hydrogel system promoted skin wound healing and increased tensile strength, thus offering new potential in the treatment of wound healing.

**Supplementary Information:**

The online version contains supplementary material available at 10.1186/s12951-024-02666-3.

## Introduction

Wound dehiscence (WD) is the partial or total separation of previously approximated wound edges, which typically occurs 5 to 8 days post-surgery when healing is still in the early stages [1]. Surgical wound dehiscence (SWD) after a previously closed surgical incision contributes to increased morbidity and mortality rates, as well as implicit and explicit costs for individuals and health care providers [2, 3]. Several surgical techniques such as tight sutures, staples, adhesive tapes or skin glues [4, 5], and negative pressure wound therapy [6, 7], as well as intelligent (strain-programmed) patches [8] have been reported to effectively reduce the incidence of SWD. All these techniques were focused on reducing the tensile strength around the wound by utilizing external forces [9], yet the development of bioactive methods that increase the tensile strength of the wound itself represents an interesting strategy [10–14].

Skin wound healing can be divided into a series of overlapping stages including coagulation/inflammation, re-epithelialization, and subcutaneous tissue formation and remodeling [15]. For clean surgical incisions, the re-epithelialization process that builds a barrier to separate deep tissue from the outer environment is accomplished in a short period of time, while the formation and remodeling of the tissue beneath the epidermis provide the capacity to prevent SWD. Soon after injury, fibroblasts migrate to the wound site, proliferate, produce extracellular matrix (ECM), and change into myofibroblasts, thus being important not only for structure restoration but also for the tensile strength of the wound. Several experiments reported that growth factors increased the tensile strength through their effects on fibroblasts. Platelet-derived growth factor (PDGF)-BB is the only recombinant growth factor approved by the United States (US) Food and Drug Administration (FDA) for the chronic treatment of diabetic neuropathic ulcers. The sustained release of PDGF-BB alone was unable to improve wound healing and tensile strength, and positive results could only be obtained by the co-delivery of PDGF-BB and transforming growth factor (TGF)-β [16]. However, PDGF-BB has the capacity to increase tendon healing and its tensile strength [17]. Other factors such as basic fibroblast growth factor, epidermal growth factor, and vascular endothelial growth factor were either used off-label outside the US or under clinical trials [18, 19]. However, the use of recombinant growth factors for wound therapy and WD prevention has not been accepted in clinical practice owing to the limitations, such as a short half-life due to the high proteolytic activity in wounds, as well as the risk of undesirable effects like the increased risk of malignance-associated PDGF-BB [20]. Therefore, novel reliable growth factors or bioactive peptides are worthy of further research.

As a polypeptide hormone secreted by the parathyroid glands, parathyroid hormone (PTH) plays a crucial role in regulating calcium deposition, collagen synthesis, and cell migration [21–23]. Currently, PTH(1–34) (teriparatide) has been approved by the FDA for the treatment of osteoporosis due to its safety and efficacy [24]. Previous studies have revealed that PTH receptors are also expressed in fibroblasts, keratinocytes, and vascular endothelial cells, indicating that PTH is a promising candidate for wound repair. However, considering the negative effects of PTH on bone metabolism when continuously and locally administrated, the local application of PTH might also have inhibitive effects on wound healing. To address this issue, we designed a novel short peptide, PTH(3–34)(29–34) or MY-1, by partially replacing and repeating the key amino acid domains of PTH(1–34). Our former study revealed that when locally administered, MY-1 demonstrated a capacity to increase skin wound healing by promoting keratinocyte migration, wound re-epithelialization, and epidermis formation [25]. At the same time, we noticed that fibroblasts were also significantly affected by MY-1. In the current experiments, we investigated its effects on fibroblasts in a more sustainable releasing system.

The development of local drug delivery systems by incorporating drugs into bio-active materials has been found to boost the drug’s effects in wound therapy [26–28]. Among the reported biomaterials, hydrogels were found to possess special merits, among which methacryloyl gelatin (GelMA) is one of the most widely accepted candidates, attributed to its injectability, good biocompatibility, mechanical performance, and biodegradability. It was utilized as a carrier for delivering growth factors, antibiotics, and other bioactive molecules. Through incorporating bioactive materials, functionalized GelMA hydrogels acquire the ability to promote wound closure by stimulating the proliferation, differentiation, and migration of restorative cells while suppressing inflammation [29, 30]. However, the drug-functionalized GelMA systems are still relatively uncontrollable in drug release (burst release over a short period of time), unstable in terms of local drug concentrations, and thus incapable of maintaining the drug bioactivity [31, 32]. To avoid its shortcomings, drugs encapsulated in liposomes and then loaded into GelMA could represent an ideal strategy that is mutually complementary. Liposomes as the nano-sized vesicles that are composed of phospholipid bilayers are currently attracting increased attention, due to the phospholipid composition being coincident with cell membranes, thus endowing them with excellent transport capacity, biocompatibility, and biodegradability. Furthermore, as carriers for peptide-based drugs, liposomes offer excellent drug stability and sustained drug release, while facilitating targeted therapy [32–35]. However, due to their liquid state, locally administered liposomes can easily leak from the wound region, resulting in challenges in terms of maintaining an efficient concentration in the wound area [32] Hence, embedding nanoliposomes into a porous hydrogel in order to limit their rapid escape could represent a promising method for wound therapy.

**Fig. 1:**
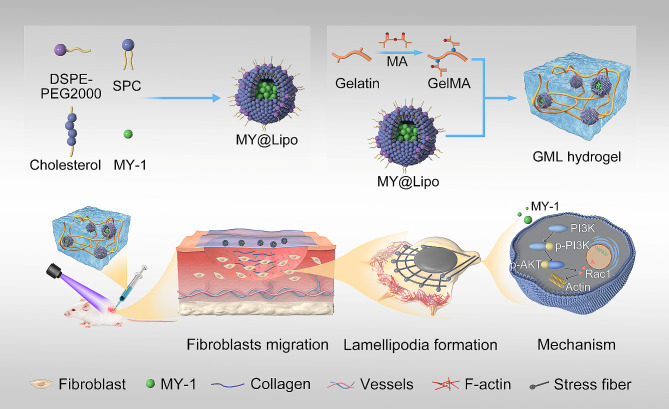
Schematic illustration of how GML hydrogel was fabricated and the mechanism by which GML promoted the wound healing

In the present investigation, we developed a nanocomposite liposome loading an MY-1-incorporated GelMA-based injectable hydrogel (GelMA–MY@Lipo, or GML), and applied it on acute full thickness cutaneous defects to observe its effect on the wound closure and the tensile strength of the wound healing. Our study revealed that the controlled release of MY-1 from GML promoted wound healing and enhanced the tensile strength by regulating fibroblast migration through activation of the PI3K/AKT-Rac1 signaling pathway in fibroblasts (Fig. 1).

## Methods and materials

### Design and synthesis of MY-1 peptide

Based on the amino acid sequence of PTH(1–34), MY-1 was designed by truncating the key amino acid domain of PTH(1–2) and duplicating the key acid domain of PTH(29–34). As previously reported, the sequence of MY-1 was SEIQLMHNLGKHLNSMERVEWLRKKLQDVHNYQDVHNY [36]. MY-1 and MY-1-FITC were synthesized with the assistance of Mecklin Co., Ltd. (Shanghai, China). The concentration and elemental composition of the MY-1 peptide were verified by mass spectrometry, confirming a purity of over 95%.

### Fabrication of MY-1-encapsulated liposomes (MY@Lipo)

Soybean phosphatidylcholine (SPC), cholesterol, and 1,2-distearoyl-sn-glycero-3-phosphoethanolamine-N-[methoxy(polyethylene glycol)-2000] (DSPE-PEG-2000) were co-dissolved in 3 mL of chloroform and subsequently evaporated under reduced pressure to create a lipid film within a sample vial. Subsequently, 0.5 mg of the MY-1 or MY-1–FITC was introduced, and the mixture supplemented with 2 mL of deionized water. Then, the solution was subjected to sonication and extruded through a liposome extruder with a polycarbonate membrane (pore size: 100 nm; Whatman, Maiterstone, England). Following this, the sample underwent dialysis utilizing a nanodialysis device equipped with a polycarbonate membrane (pore size: 30 nm) to eliminate any unencapsulated peptides. Finally, the volume was adjusted to 3 mL with deionized water and subjected to lyophilization.

### Characterization of MY@Lipo

The hydrodynamic diameter of fresh liposomes was detected by dynamic light scattering (DLS). The polydispersity index (PDI) and zeta potential were measured by the phase analysis of light scattering (PALS) with the NanoBrook 90 plus PALS particle size and zeta potential analyzer (Bruker, Berlin, Germany). The morphology and stability of MY@Lipo and empty liposomes was evaluated by transmission electron microscope (TEM) (TF20; FEI, Hillsboro, USA).

The encapsulation efficiency (EE) and drug loading (DL) capacity were obtained via the ultraviolet-visible (UV–Vis) absorbance of MY–FITC (as an indicator) using the absorbance method. The EE (%) of MY@Lipo was calculated via the following equation: EE (%) = (the amount of total peptide – the amount of free peptide) / the amount of total peptide × 100%. Meanwhile, the DL (%) of MY@Lipo was calculated utilizing the following formula: (the amount of total peptide – the amount of free peptide) / the total weight of MY@Lipo × 100%.

### Preparation and characterization of GelMA/GML hydrogels

5% (w/v) GelMA hydrogels were prepared according to the manufacturer’s instructions (EFL-Tech Co., Ltd. Suzhou, China) and our previous report [25]. Briefly, 1 g of GelMA was completely dissolved into 20 ml of 0.25% lithium phenyl-2,4,6-trimethylbenzoylphosphinate solution at 65 ℃ for 30 min. Then, the solution was filtered via a 0.22 μm filter (Millipore, Shanghai, China) and stored at 4 ℃ in a dark location for further experiments. MY@Lipo was added to the GelMA solution at 37 ℃ (GML hydrogel) and cross-linked for 40 s by ultraviolet light (365 nm wavelength, 5 watts power) immediately or stored at 4 ℃ in a dark location for further usage.

### Morphology and structural characterization of hydrogels

The morphologies of the crosslinked GelMA and GML hydrogels were observed with a field emission scanning electronic microscope (FE-SEM) (Sigma 300; Carl Zeiss). The diameter of the pores was measured. Energy dispersive spectrometry (EDS) analysis was subsequently carried out to assess the elemental composition of the two types of hydrogels. To observe the distribution of MY@Lipo in the hydrogels, the liposomes were first labeled with Dio (Beyotime, Shanghai, China) for 30 min and then incorporated into the hydrogel. After 40 s of ultraviolet irradiation, the distribution of liposomes in the GML–Dio hydrogel was observed by confocal microscope (LSM980, Carl Zeiss, Oberkochen, Germany) and the 3D images were subsequently reconstructed. The chemical composition of the GelMA and GML was characterized by Fourier transform-infrared spectroscopy (FTIR) (Vector 33; Bruker, Berlin, Germany) with the wavelength in the 500–4000 cm^− 1^ range and a 4 cm^− 1^ resolution.

### Swelling and degradation assay

To detect the swelling character of the hydrogels, the GelMA and GML were lyophilized and weighed as M(0). The hydrogels were immersed in PBS and placed in an incubator at 37 ℃. At the time points of 1, 3, 6, 12, 24, and 36 h, the water outside the hydrogels was removed and the weights of the swelling hydrogels were measured as M(1). The swelling ratio of each time point was calculated using the following equation: swelling ratio (%) = (M(l) – M(0)) / M(0) × 100%.

For the degradation assay, hydrogel samples (100 µL) were first prepared into cylinders and cross-linked with ultraviolet light. The samples on day 0 were lyophilized, and their weights were recorded as W0. Identical volumes of hydrogel samples were immersed in PBS and incubated at 37 °C. At each time point, samples were removed from the PBS, lyophilized, and weighed to determine the remaining weight (W1). The degradation rate at each time point was calculated using the following equation: degradation ratio (%) = (W1 / W0) × 100%.

### MY-1 release assay

For the in vitro MY-1 release assay, a standard curve was generated to correlate the optical density (OD) value of the FITC with graded concentrations of MY-1–FITC. Then, the GML–FITC was cross-linked, immersed in 10 mL PBS in centrifuge tubes, and placed in a shaker at 37 °C in a dark location. The 100 µL supernatants were collected at a certain time point, and an equal volume of PBS was added to the system. The OD values of the supernatants were measured via the Spectra Max i3x multi-mode microplate reader (Molecular Devices Co., Ltd., San Jose, CA, USA) at an excitation wavelength of 488 nm, and subsequently entered into the formula of standard curve to calculate the concentrations of MY-1 at each time point. An in vivo peptide releasing test was carried out on rat skin wound models, with the fluorescence intensities and locations detected by an in vivo imaging system.

### *In vitro * and *in vivo* biocompatibility analysis

For in vitro biocompatibility testing, GML containing serial concentrations of MY-1(0, 0.01, 0.1, 1, or 10 µg per 100 µL gel) was generated and placed in the upper chamber of an 8 μm pore-size transwell plate, then the primary rat dermal fibroblasts were cultured in the lower chamber of the plate in DMEM medium that submerged the hydrogel. After 24 h, the primary rat dermal fibroblasts were stained utilizing a Calcein-AM/PI kit (Beyotime) according to the manufacturer’s instructions. Living cells exhibited green fluorescence and dead cells were stained in red. In vivo biocompatibility testing was conducted on rat skin wound models with hydrogels carrying different concentrations of MY-1, as mentioned above, with the in vivo biocompatibility demonstrated by the wound width at day 7.

### Animal experiments

Male SD rats (SPF, 250 ± 30 g) were purchased from the Animal Center of Southern Medical University, Guangdong Province, China. All animal experiments involved in this study strictly complied with the guidance for the care and use of laboratory animals, and the protocols were approved by the Ethical Committee of the Laboratory Animal Center of Southern Medical University (Grant No. NFYY-2022-0417). The rats were housed under standard temperature and humidity conditions, with a 12 h light/dark cycle and free access to food and water. Before surgery, all rats were anesthetized by gaseous anesthesia with the inhalation of isoflurane, and the dorsal hairs were shaved with a razor and further depilated with depilatory cream. Then, the dorsal skin was sterilized with alcohol-impregnated gauze and two symmetric full-thickness skin wounds (diameter: 15 mm) were created on the back of animals with the help of a puncher and scissors. In order to homogenize the wound contraction and mark the edge, a rubber ring was sewn around the skin wound.

The in vivo experiments were divided into three stages. In the first stage, the rat models were randomly subdivided into six groups and the wound was filled with 100 µL GelMA (group Gel), or GML groups with MY-1 at 0.1–10 µg. In the second stage, the rat models were randomly subdivided into four groups and the wound was filled with 100 µL of PBS (group Blank), GelMA (group Gel), GelMA–liposome (group GL), or GelMA–MY@Lipo (group GML). In the third stage of the in vivo experiment, a PI3K/AKT signaling inhibitor, LY294002 ([LY]; Beyotime), was loaded into the GelMA at 100 µg per wound. The rat models were randomly subdivided into four groups and the skin defects were filled with GelMA–Lipo, GML, GelMA–Lipo–LY, or GML–LY, respectively. After that, all skin wounds were overlaid by Tegaderm™ film (3 M Health Care, St. Paul, MN, USA) and protected with gauzes and bandages. The skin wounds were photographed at certain periods of time post-operation. The wound areas at day 0 and the other time points were measured with the Image J software (recorded as A0 and At, respectively). The remaining wound area was calculated as a percentage using the following formula: remaining wound area = At / A0 × 100%. At days 3, 7, and 14, the animals were euthanatized, and the dorsal skin samples were harvested and fixed in 4% paraformaldehyde (Leagene, Beijing, China).

### Tensile strength measurement

At day 14, the rat models were euthanized. Centered on the wound, fresh full-thickness rectangular skin samples sized 1 cm × 0.5 cm were isolated. Then, the samples were fixed on the tensiometer of an electronic universal testing machine (Aoke Testing Instrument Co., Ltd. Guangzhou, Guangdong Province, China), at both ends along the long axis. The stretch power was applied evenly along the short edge of the rectangular skin samples and the stretch speed was set at 10 mm/min. The highest power prior to the breakage was defined as the tensile strength of each sample.

### Histological analysis

After being fixed in 4% paraformaldehyde for 24 h, the skin samples were dehydrated, embedded, and sectioned following a standard protocol. Four-micrometer-thick sections were dewaxed and processed with a hematoxylin-eosin (HE) staining and Masson’s trichrome staining. For the exhibition of collagen, the samples were cut into 6-µm-thick sections and dewaxed, followed by immersion in picrosirius red for 1 h at room temperature. The sections were then washed twice in water, stained with Mayer hematoxylin for 8 min, and rapidly dehydrated in xylene. The depositions of collagen I and collagen III fibers were detected by light and polarized light microscopy (BX53P; Olympus, Tokyo, Japan). The images were analyzed utilizing the Image J software, as previously described.

For immunohistochemical (IHC) staining, the sections were dewaxed and heated in a microwave for antigen retrieval. After that, the sections were immersed in 3% H_2_O_2_ for 15 min, followed by incubation in goat serum for 30 min at room temperature to block the antigen. Subsequently, all sections were incubated in primary antibodies against fibronectin ([FN]; dilution at 1:250; Affinity Biosciences, Beijing, China), Rac1 (dilution at 1:250; Santa Cruz Biotechnology, Cincinnati, OH, USA), p-AKT (dilution at 1:250; Affinity Biosciences) and p-PI3K (dilution at 1:250; Affinity Biosciences) overnight at 4 ℃. Then, the sections were incubated with a second antibody for 2 h at room temperature. The histological images were developed with a DAB system (ZSGB-Bio, Beijing, China) and investigated via a BX63 microscope (Olympus).

For immunofluorescence (IF) staining, the 4-µm-thick sections were subjected to dewaxing and antigen retrieval, followed by 15 min of permeabilization with 0.5% Triton X-100 (Solarbio, Beijing, China). Primary antibodies against Ki67 (dilution at 1:250; Affinity Biosciences) and Vimentin (dilution at 1:250; Santa Cruz) were utilized. Then, the sections were incubated in Alexa Fluor 488- or Alexa Fluor 594-conjugated secondary antibodies (Abcam, Cambridge, UK) at room temperature for 2 h, and the nuclei stained with 4,6-diamidino-2-phenylindole dilactate ([DAPI]; Beyotime). The images were visualized using the LSM-980 confocal microscope.

### Cell counting kit-8 assay and colony forming unit assay

The cell viability was detected utilizing a cell counting kit-8 ([CCK-8]; Dojindo, Kumamoto, Japan). Briefly, fibroblasts were placed into 96-well plates at a density of 2 × 10^3^ cells per well. Serial concentrations of MY-1 in the 1–10 µM range were added to the culture system. After 24–72 h, the medium was removed and 100 µL serum-free DMEM containing 10 µL CCK-8 solution was added, with the solution then incubated at 37 ℃ for 1 h. The OD at the wavelength of 450 nm was measured via the Molecular Devices Spectra Max i3x multi-mode microplate reader.

Primary fibroblasts of the third passage were seeded in 6-well plates at a density of 1000 cells per well. Seven days later, the cells in different group were fixed with 4% PFA and stained with 0.1% crystal violet (Leagene) for 15 min. Cell colonies containing 50 or more cells were selected and counted.

### Cell migration assays

For the cell scratching assay, fibroblasts were seeded in 12-well plates at a density of 4.5 × 10^5^ per well. Prior to scratching, the cells were pre-stimulated with 10 nM or 100 nM MY-1 or the control for 24 h. When the cells reached 100% confluence, a sterile yellow pipette tip was inserted until it firmly touched the base of the culture dish, and then it was moved from one side to another while remaining vertical. Next, the floated fibroblasts were removed by washing with PBS. At certain time points, the cells were fixed with 4% paraformaldehyde and then stained with rhodamine-phalloidin (Abcam) for 30 min, followed by staining with DAPI for an additional 10 min. Images were taken with an inverted fluorescence microscope (IX73; Olympus). The difference in cell migration ability was defined as the percentage of the cells-recovered area compared to the original scratched area.

Transwell assay was performed to demonstrate the cell motility. The primary fibroblasts were pre-treated with 10 nM or 100 nM MY-1 or vehicle for 24 h prior to being subjected to the transwell assay. A total of 5 × 10^4^ fibroblasts suspended in 100 µL serum-free medium was seeded into the upper chamber of a 24-well, 8 μm pore-size transwell plate (Corning, NY, USA). The culture medium containing 2% FBS with or without MY-1was added into the lower chamber. Following 48 h of incubation, the cells remaining on the upper side of the chambers were swabbed with cotton swabs, while the migrated cells growing on the lower side of the chambers were fixed in 4% paraformaldehyde for 15 min and stained with 0.1% (w/v) crystal violet (Leagene) for an additional 15 min. Images of the stained cells were collected via an inverted phase-contrast microscope (IX73; Olympus), and the number of cells were quantified.

Wound explant assays were employed to assess the migrating ability of cells from the tissue. The dermis was isolated from the dorsal skin of 1-day-old SD rat neonates. Briefly, the skin patches were harvested and digested with 0.1% dispase (Solarbio) to separate the dermis from epidermis. The collected dermis was cut into small pieces (diameter: 1 mm) with scissors and placed into a well that was pre-coated with collagen type I. After incubation with 10% FBS-supplemented medium containing peptides or other agents for 5 days, the cells sprouting from the dermis tissue were investigated by invert microscope (IX73; Olympus). The distance from the dermis tissue to the farthest sprouting edge was measured and normalized as the fold of control (set as 1.0).

### Cytoskeleton staining

For cytoskeleton and lamellipodia staining, fibroblasts were seeded in the 24-well glass cover slips at a density of 1 × 10^4^ per well. After adhesion, the complete medium was exchanged with a DMEM medium (with 2% FBS) with or without MY-1, and special inhibitors. Then, the fibroblasts in each group were fixed with 4% PFA for 15 min and stained with rhodamine-phalloidin for 30 min, followed by 10 min of DAPI staining. The cell morphology and lamellipodia were observed using the confocal microscope. The amount of lamellipodia was defined as the percentage of lamellipodia area compared to the total cell area.

### Immunofluorescence staining of fibroblasts

The fibroblasts were fixed with 4% paraformaldehyde for 15 min at room temperature, followed by permeabilizing with 0.5% (v/v) Triton X-100 (Solarbio) in PBS for 15 min. After washing with PBS, the cells were blocked in 5% BSA (Solarbio) for 1 h and incubated in primary antibodies overnight at 4 °C. Subsequently, the cells were washed again and incubated in Alexa Fluor 488- or 594-conjugated secondary antibodies for 2 h at room temperature. Finally, DAPI was utilized to stain the nuclei for 10 min. Fluorescence images were observed by confocal laser scanning microscopy.

### Western blot analysis

The total protein was extracted utilizing the RIPA lysis buffer (Beyotime) with protease inhibitor (Beyotime) and protein phosphatase inhibitor (Beyotime) on ice. The cell lysates were then collected and subjected to ultra-sonication for 10 min, followed by 15 min of centrifugation (4 ℃, 12,000 rpm) and protein concentration measurement with the BCA protein assay kit (Beyotime). Equal amounts of protein (30 µg per lane) were subjected to 10% (w/v) SDS-PAGE and transferred to a polyvinylidene fluoride (PVDF) membrane (Millipore). After blocking with the 5% BSA, the PVDF membrane was incubated with primary antibodies against GAPDH (dilution at 1:1000; Affinity Biosciences), Rac1 (dilution at 1:1000), PI3K (dilution at 1:1000), AKT (dilution at 1:1000), p-PI3K (dilution at 1:1000), or p-AKT (dilution at 1:1000) at 4 °C overnight. The membranes were then washed with TBST (Beyotime), and incubated with the corresponding HRP-conjugated secondary antibodies for 2 h at room temperature. The proteins were visualized through chemiluminescence and imaged on an enhanced chemiluminescence agent (BLT Co., Ltd., Guangzhou, China). The protein bands were quantified by densitometry analysis via the Image J software.

### siRNA transfection

siRac1 and negative control siRNA were synthesized by Qingke Co., Ltd. (Beijing, China). siRNA transfection was carried out when the fibroblasts reached 60–80% confluence using Lipo3000 Transfection Reagent (Invitrogen, Carlsbad, CA, USA) according to the manufacturer’s instructions.

### Statistical analysis

All quantitative data were expressed as mean ± standard deviation (SD) from at least three repetitive experiments or six animals. For the analysis of two groups, the unpaired Student’s t-test was employed for normally distributed data, while the Mann–Whitney U test was applied for non-normally distributed data. The one-way ANOVA and Tukey’s post hoc test were utilized to determine the significance of difference with SPSS (v.21.0) software (IBM SPSS Inc., Chicago, IL, USA), where a value of *P* < 0.05 was considered statistically significant.

## Results

### Design of MY-1, characterizations of MY@Lipo, and determination of the appropriate concentration in hydrogel

**Fig. 2:**
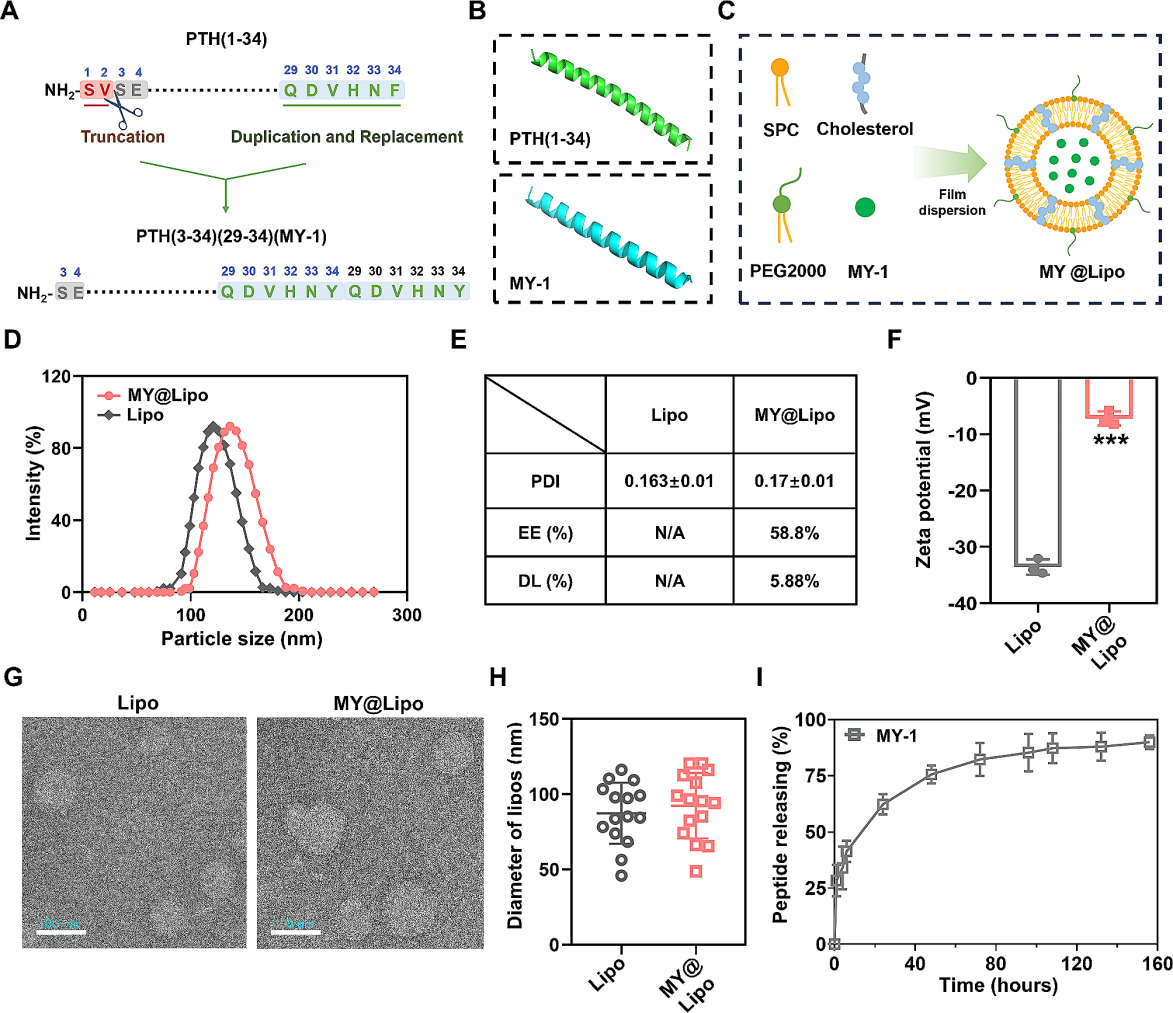
Design of MY-1 and characterization of the liposomes. Schematic illustration of how MY-1 was designed **(A)**. The three-dimensional structure of MY-1 and PTH(1–34) was reconstructed using Alphafold 3 software (B). Schematic illustration of the MY-1 encapsulation process into liposomes **(C)**. The hydrodynamic diameters of the liposomes with or without MY-1 **(D).** The dispersity index (PDI), encapsulation efficiency (EE), and the drug loading capacity (DL) of the liposomes with/without MY-1 **(E)**. Zeta potential of the liposomes with/without MY-1 (**F**). SEM images and diameters of the liposomes with or without MY-1 **(G** and **H)**. MY-1 was encapsulated into liposomes and immersed in PBS, the peptide releasing speed was calculated by the amount ratio of MY-1 in PBS over MY-1 in total at a certain time point (**I)**. Data are expressed as mean ± SD. The experiments were repeated three times with similar results. ***, *p* < 0.001. Scale bars: 100 nm in (G)

The design diagram of MY-1 was shown in Fig. 2A. The spatial structure of PTH(1–34) and MY-1 were predicted with AlphaFold 3. As per the results, both PTH(1–34) and MY-1 were short peptides with simple structures featuring several classic α-helices (Fig. 2B). MY@Lipo was synthesized utilizing SPC, cholesterol, DSPE-PEG-2000, and MY-1 through the film dispersion-ultrasonication method (Fig. 2C). Meanwhile, the liposome without MY-1 loaded was also synthesized as the control. As shown in Fig. 2D, two types of liposomes had similar hydrodynamic diameters: 134.00 ± 2.00 nm in the MY@Lipo group and 133.33 ± 3.79 nm in the empty liposomes. The PDI of MY@Lipo and empty liposome were 0.170 ± 0.01 and 0.163 ± 0.01, respectively (Fig. 2E), indicating that both types of nanoparticles had a similar size. The EE (%) and DL (%) of MY@Lipo as measured by UV–Vis spectrometry were 58.8% and 5.8%, respectively (Fig. 2E), suggesting that a reasonable loading efficiency had been achieved. Meanwhile, the zeta potential of the two types of liposomes was measured. As per the results in Fig. 2F, the zeta potential of MY@Lipo was − 7.207 ± 1.26 mV, and larger than the − 33.590 ± 1.37 mV for the liposome particles, which was attributed to the positive charge carried by MY-1. The morphology of the MY@Lipo and empty liposomes was observed by TEM, which showed that both MY@Lipo and empty liposome were dispersive spheres with diameters ranging from 50 to 120 nm (Fig. 2G and 2H). Notably, the diameters of liposomes in the TEM images were smaller than those detected by DLS, likely due to the particles being hydrated during the particle size detection. The results of TEM showed that both empty liposomes and MY@Lipo retained their diameters and spherical morphology after 7 days of water immersion, suggesting that the liposomes possessed stability. (Supplementary Fig. 1A and 1B). The drug release assay showed that MY-1 experienced a quick release from liposomes that occurred in the first 6 h, followed by a slow released in the following days, suggesting that to some extent, the liposomes retained the peptide and avoided the rapid peptide diffusion.

To generate a sustained MY-1 release system, the MY@Lipo was mixed in the GelMA solution, and after ultraviolet crosslinking, the GML gel containing a serial amount of MY-1 was co-cultured with the primary dermal fibroblasts (Supplementary Fig. 2A). A suitable amount of MY@Lipo for the fibroblasts culture was detected by live/dead staining, which showed that certain concentrations of encapsulated MY-1 (0.01 to 10 µg/mL) did not exhibit cytotoxicity on fibroblasts, compared to the increased number of dead fibroblasts at higher MY-1 concentration (100 µg/mL) that presented (Supplementary Fig. 2B and 2D). Similar results were also detected in the in vivo study, with the inhibition of wound healing at 100 µg/mL, while positive effects were observed at 1 µg/mL (Supplementary Fig. 2C and 2E). Therefore, GML containing 1 µg/mL MY-1 represented a suitable concentration for increasing wound healing.

### Characterizations of the GelMA and GML hydrogels

**Fig. 3:**
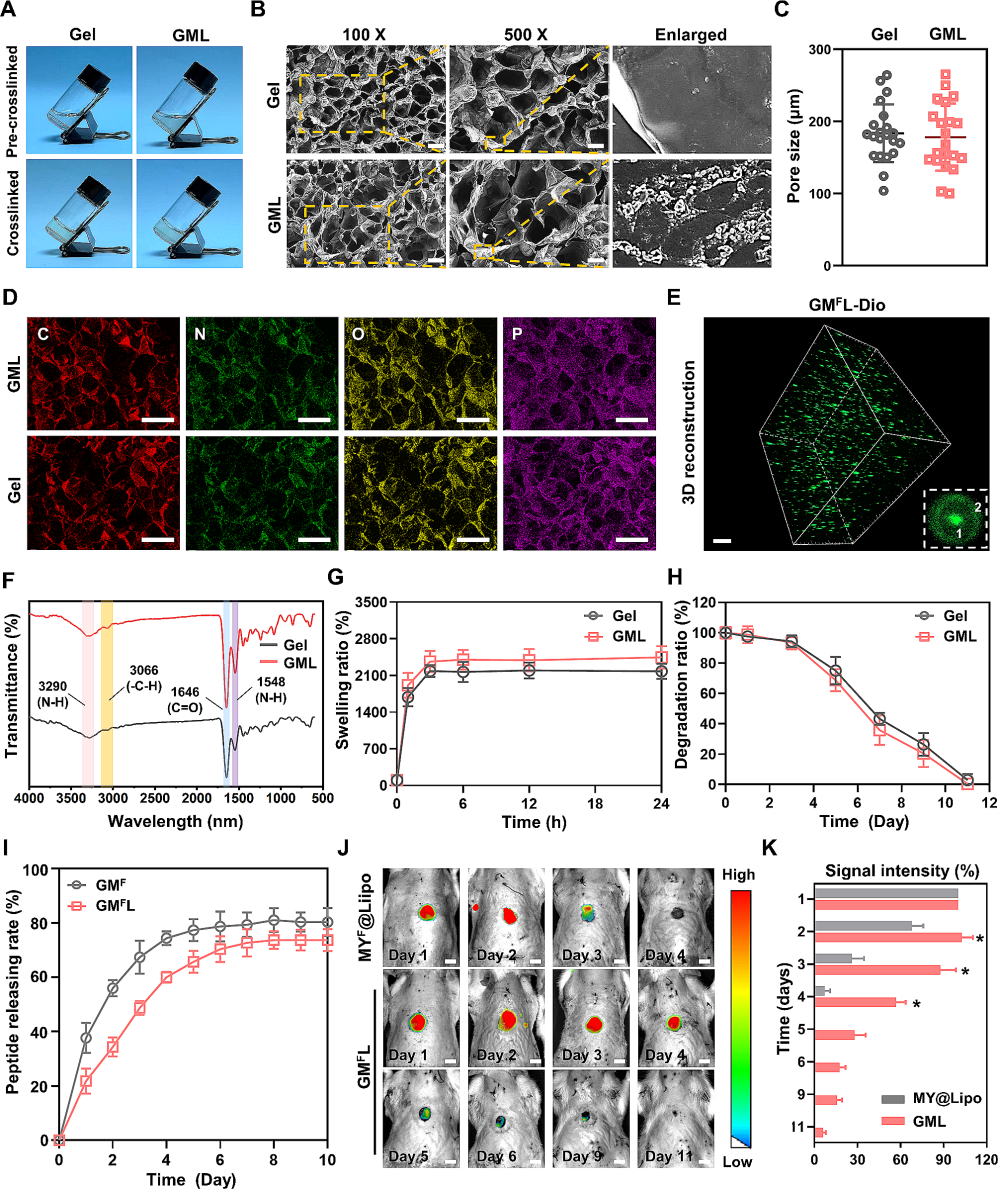
Characterization of the GelMA and GML hydrogels. The gelation of GelMA (Gel) and GML hydrogels after exposed to the ultraviolet ray for 40 s **(A)**. The microstructure of GelMA and GLM was investigated by SEM (**B)**. Pore size of Gel and GMLwas measured base on SEM images (**C)**. The Chemical elements of carbon (C), nitrogen (N), oxygen (O) and phosphorus (P) in Gel and GML were analyzed by EDS spectrum **(D)**. 3D views of MY-1-FITC in the Dio-labeled liposomes (MY^F^@Lipo-Dio) in the GelMA scaffold was evaluated using a confocal microscope. Dio (green) was used to label the member of liposomes (Symbol ‘2’ in the upper panel, green), and FITC was used to characterize the MY-1 in the core (Symbol ‘1’ in the upper panel, green) **(E)**. FTIR spectroscopy was carried out to detect the characteristic peaks of Gel and GML **(F)**. In vitro swelling was quantified by the weight ratio of hydrogels maintained in PBS at 37 ℃ versus its freeze-dried weight at time zero **(G)**. Gel and GML hydrogels were immersed in PBS at 37 ℃, the degradation properties of the materials were presented by the ratio of remaining to the original amount of the freeze-dried hydrogels at time zero (**H)**. MY^F^@Lipo and then incorporated into GelMA (GML^F^) and immersed in 10 mL PBS, the peptide releasing speed was calculated by the amount ratio of FITC in PBS over FITC in total at a certain time point **(I)**. Full-thickness skin wounds with a diameter of 15 mm were applied with MY^F^@Lipo or GML^F^ (**J**) and the intensity of FITC was measured (**K)**. The remaining FITC presented the amount of peptide in the wound. Data were expressed as mean ± SD. The experiments were repeated three times with similar results. *, *p* < 0.05. Scale bars: 200 μm and100 μm in (B), 200 μm in (D), 10 μm in (E) and 5 mm in (J)

The GelMA hydrogel exhibited a transparent, smooth, and colorless appearance, while the incorporation of MY@Lipo slightly reduced the transparency. However, both GelMA and GML could be successfully crosslinked after 40 s of ultraviolet irradiation (Fig. 3A). The microstructures of GelMA and GML were examined by SEM, with both hydrogels presenting with a similar porous appearance (Fig. 3B and 3C). Interestingly, the inner pore surface inside the GelMA was much smoother, compared to a significantly rougher inner wall after the incorporation of MY@Lipo (Fig. 3B and 3C). The chemical elements of the hydrogels were analyzed by EDS spectrum. Similarly, the elements of carbon, nitrogen, and oxygen in the GelMA and GML were notable but showed little difference, which could be attributed to the relatively low amount of MY@Lipo loaded in the GML (Fig. 3D and Supplementary Fig. 3). Notably, the phosphorus element, which is abundant in liposomes, was also detected in the GelMA hydrogel (Fig. 3D and Supplementary Fig. 3). This phenomenon might be attributed to the usage of PBS and LAP during the preparation of GelMA. To evaluate the distribution and morphology of MY@Lipo in the GelMA hydrogel, MY–FITC-loaded liposomes (MY^F^@Lipo) were first labeled with Dio and then incorporated into the hydrogel. The image of 3D analysis showed that the spherical liposomes were uniformly distributed in the hydrogel (Fig. 3E). In the single MY^F^@Lipo–Dio, the MY-1–FITC was retained in the core of the liposomes (Fig. 3E, symbol ‘1’, green), while the phospholipid bilayers of liposomes were labeled with Dio (Fig. 3E, symbol ‘2’, green). FTIR spectroscopy was carried out to detect the characteristic peaks of GelMA and GML, with the results revealing that the classic peaks obtained from the GelMA, the N-H stretching (amide A) at 3290 cm^− 1^, C = O stretching (amide I) at 1646 cm^− 1^, and N-H bending (amide II) at 1540 cm^− 1^ were detected in both types of hydrogels (Fig. 3F). Additionally, -CH at 3066 cm^− 1^ was detected in the GML hydrogel, which was considered to be associated with the incorporation of liposomes (Fig. 3F) [37]. However, we were unable to detect the other characteristic peaks for MY@Lipo in the GML, which might be attributed to the low amount of MY@Lipo in the GML hydrogel or the low sensitivity of the FTIR [38].

The swelling properties of the hydrogels revealed that both GelMA and GML reached swelling equilibrium after 3 h in PBS, and that the swelling ratio of GML (2447.12%) was slightly higher than that of GelMA (2178.12%), but there was no statistical significance (*P* > 0.05) (Fig. 3G). When comparing the degradation capacity, the GelMA and GML hydrogels displayed similar degradability, with approximately 50% of the weight remaining at day 7 and none remaining at day 11 (Fig. 3H). Collectively, GelMA and GML exhibited similar swelling and degradation characteristics, indicating that the incorporation of MY@Lipo did not change the chemicophysical properties of GelMA.

The in vitro peptide releasing measurement showed that the MY-1–FITC in the liposomes was released comparatively slowly in GelMA, as compared with the MY-1–FITC alone in GelMA, and then gradually reached a releasing balance from then onwards (Fig. 3I). The in vivo peptide releasing assay showed that the fluorescence signals in the skin wounds exhibited a two-phase release style with a quick release from day 1 to day 4, and then a gradual release from day 5 to day 9; when MY@Lipo alone was applied to the wound, the peptide signal quickly disappeared in the first 4 days (Fig. 3J and K). Thus, the GelMA/liposome system successfully controlled the local releasing of MY-1.

### GML accelerated skin wound healing and enhanced the tensile strength

**Fig. 4:**
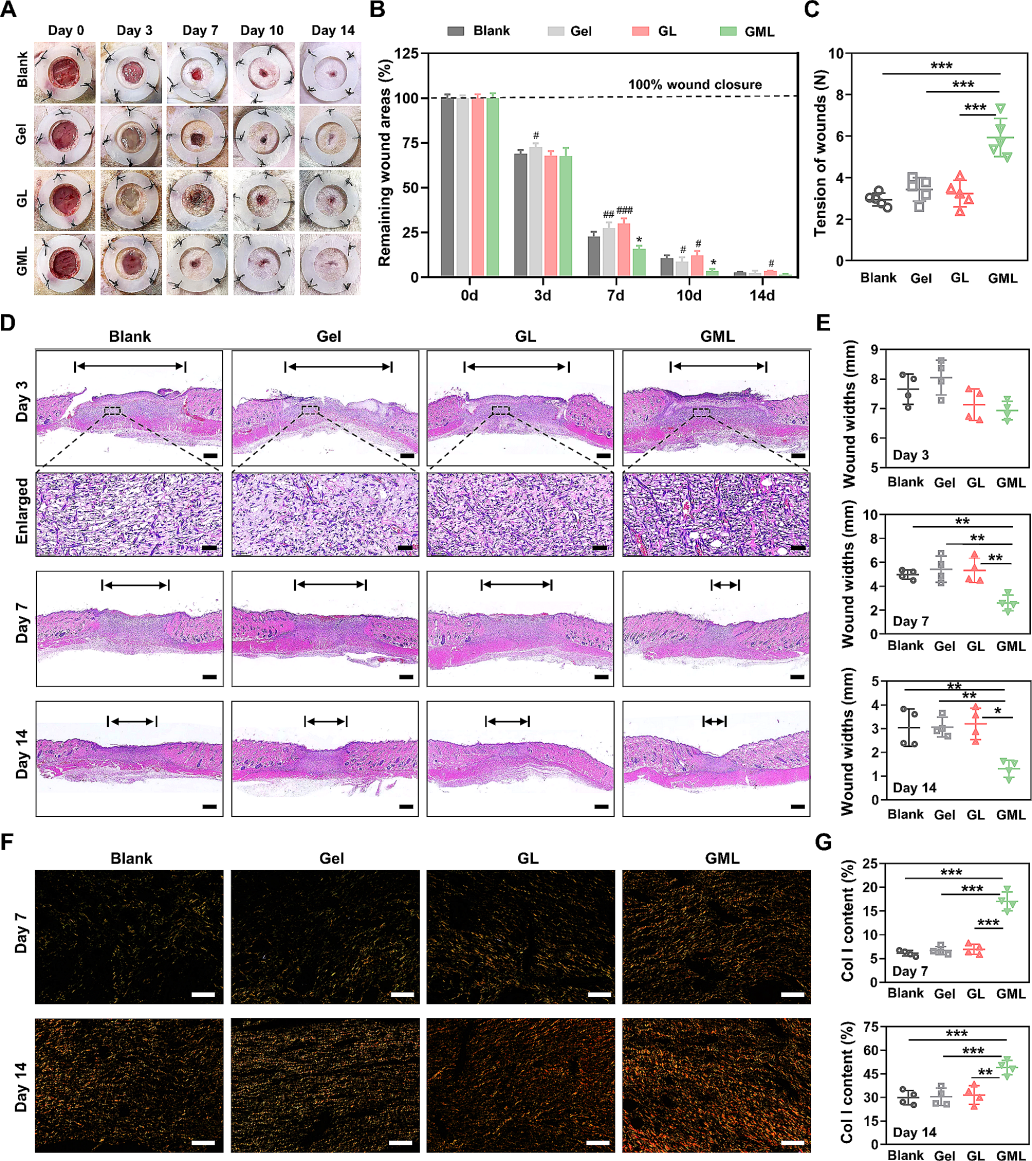
Application of GML hydrogel accelerated skin wound healing and increased tensile strength. Full-thickness skin wounds with a diameter of 15 mm were generated on the back of SD rats. PBS, Gel, GL, or GML were applied into the defects and then covered with sterilized dressing. Wound closure was investigated and photographed at certain time point as indicated (**A**). The unclosed wound area at each time point of four treatments were quantified (**B**). The tensile strength of the wounds treated by the above treatments were also measured as indicated in the *material and methods* section (**C**). Microscopic structure was demonstrated by histological sectioning and HE staining (**D**) at day 3, 7 and 14. The wound width (day 3, day 7 and 14) were quantified (**E**). Components of collagen were demonstrated by Picrosirius red staining and investigated by polarized light microscopy at day 7 and 14 (**F**), and the deposition of type I collagen was quantified and presented as percentage of collagen I in all collagen components (**G**). Data were expressed as mean ± SD. The experiments were repeated three times with similar results. *, *p* < 0.05; **, *p* < 0.01; ***, *p* < 0.001 versus Blank group. #, *p* < 0.05; ##, *p* < 0.01; ###, *p* < 0.001 versus GML group. Scale bar: 1 mm and 50 μm in (D), 200 μm in (F)

To evaluate the effects of GML on the wound healing process, SD rats were grouped and the skin wounds were subjected to the PBS, GelMA, GL, and GML treatment. From day 3 to day 14, the wounds post-GML application were the fastest to close, followed by those wounds in the GelMA group, the Blank group, and finally the GL group (Fig. 4A). At day 7, 84.03% of the wound had closed after GML treatment, compared with 77.18%, 72.50%, and 70.04% following the Blank, Gel, and GL treatments, respectively. At day 10, 96.49% closure was observed after GML, compared to 89.37%, 91.23%, and 87.83% following the Blank, Gel, and GL treatments, respectively (Fig. 4B). These results indicated that introducing the MY-1 in hydrogel could accelerate skin wound healing.

The tensile strength measurement revealed that 14 days after different treatments, the maximal tension that the healing wound skin could tolerate was 2.94 N in the Blank group, 3.03 N in the GelMA group, 3.03 N in the GL group, and 5.94 N in the GML group. These results demonstrated that the application of GML hydrogel significantly improved the tensile strength of the wounds (Fig. 4C, Supplementary Fig. 4A–4C).

### GML accelerated cells aggregation in wounds and induced ECM deposition

Histological changes revealed that the width of wounds (especially at day 7 and day 14) in the GML group was narrower than that in the other groups (Fig. 4D and 4E). On day 14, numerous hair follicles were observed in the newly formed tissues of the GML group, indicating that GML promoted wound healing without scar formation (Fig. 4D and 4E). Moreover, a greater number of cells were detected in the granulation tissue 3 days post-GML application (Fig. 4D), suggesting that GML accelerated cells aggregation in the early stage of repair. Since the ECM deposition (especially the type I collagen) was the key step for skin wound repair and tensile strength increase, we next compared the collagen deposition among the four groups on day 7 and day 14. Picrosirius red staining showed more abundant collagen I (red) and less density of collagen III (green) deposition after GML treatment, implying that GML treatment promoted collagen deposition and accelerated its maturation (Fig. 4F and 4G). Similar to the picrosirius red staining, Masson’s trichrome staining revealed that the GML group had a notably higher content of collagen. At day 14, the amount and organization of collagen became similar to normal skin in the GML group (Supplementary Fig. 5A and 5B), further confirming that GML facilitated wound healing without scar formation. IHC staining showed that the GML treatment significantly increased the intensity of FN (Supplementary Fig. 5C and 5D), an important component of ECM indicated for scarless skin healing [39]. Collectively, these results demonstrated that GML accelerated skin wound healing through increasing the number of cells, as well as inducing ECM formation.

### MY-1 induced ECM deposition by enhancing fibroblast migration instead of proliferation or differentiation

**Fig. 5:**
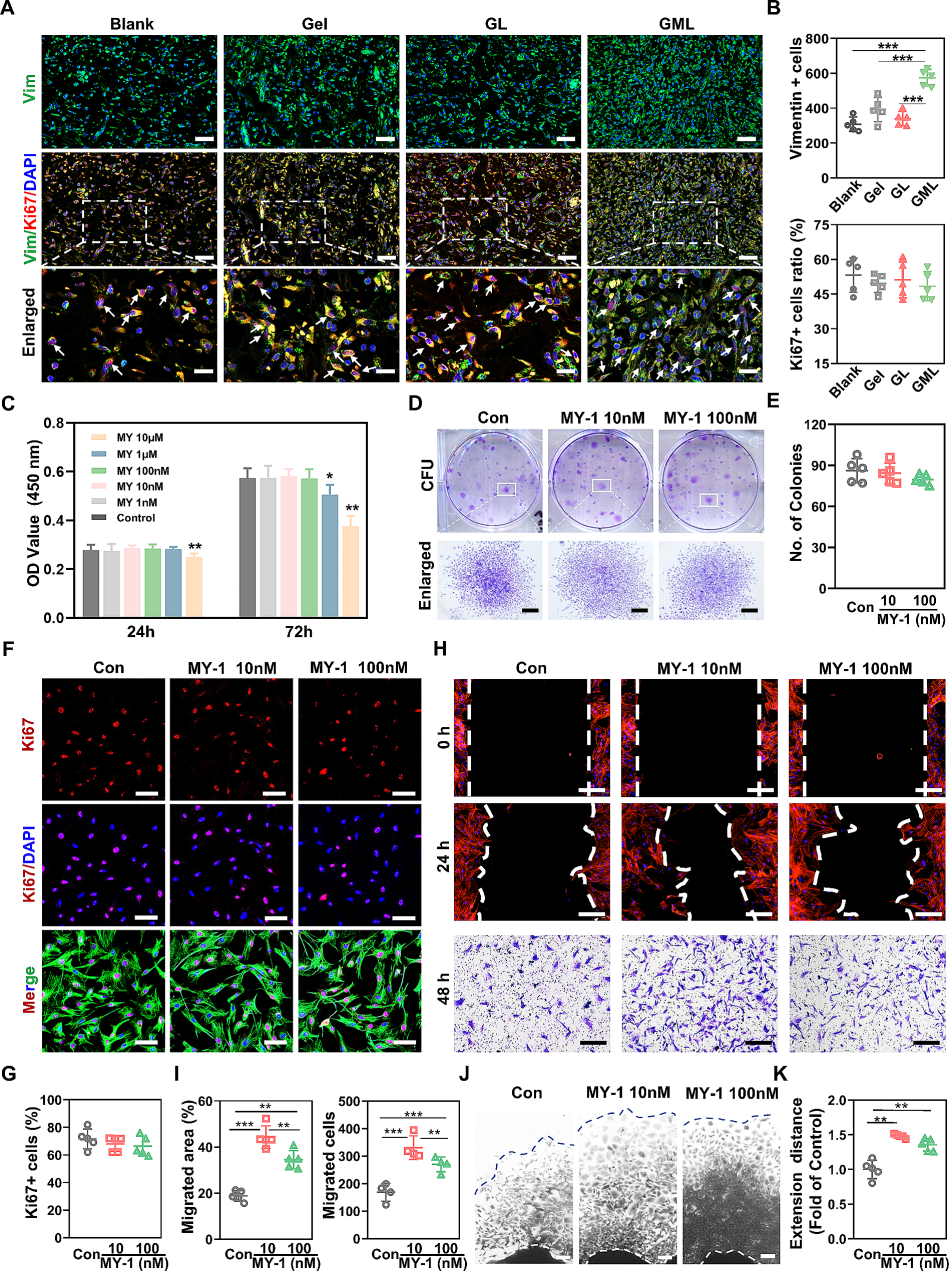
MY-1 enhanced the motility of fibroblasts in vivo and in vitro. Histological sections of skin wounds were prepared as described in Fig. 3. Expressions of Vimentin and Ki67 were demonstrated by IF staining **(A)**. The nucleus was stained with DAPI. The number of Vimentin+ cells (fibroblasts) were quantified (**B**, upper panel). The ratio of K67+  and Vimentin+ fibroblasts to Vimentin+  fibroblastspresented the proliferation portion of fibroblasts (**B**, lower panel). Fibroblasts proliferation was analyzed by CCK-8 assay (**C**) and colony formation units counting (**D and E**). Expressions of Vimentin and Ki67 in fibroblasts were also demonstrated by IF staining (**F**) and quantified as described above (**G**). Cell migration was demonstrated by cell scratching test (**H**, upper panels) and transwell assay (**H**, lowest panel). The area that refilled with migrated cells were measured and calculated to the proportion of originally scratched cell free area (**I**). The cells that migrated crossed the apertures were counted (**I**). The cells that grew and extended from dermal patches after 5 days of MY-1 addition were visualized (**J**) and quantified (**K**) as described in *materials and methods*. Data were expressed as mean ± SD. The in vitro experiments were repeated three times with similar results. *, *p* < 0.05; **, *p* < 0.01; ***, *p* < 0.001. Scale bars: Scale bars: 50 μm and 25 μm in (A), 500 μm in (D), 100 μm in (F), 200 μm in (H), and 150 μm in (J)

Since wound healing and tensile strength are directly related to ECM deposition, we next investigated the mechanisms by which GML increases ECM deposition. Given that ECM, especially collagen, is synthesized by fibroblasts, we focused on the effects of MY-1 on fibroblasts. Generally, both an increase in the number of fibroblasts (via proliferation or migration) and an enhancement in their collagen synthesis capacity contribute to the wound healing process. Considering the cells aggregation observed in HE staining (Fig. 4D), we firstly evaluated the number of fibroblasts and their proliferation capacity across the four groups by assessing the expression of Vimentin, a marker of fibroblasts, and Ki67, a marker of proliferating cells. The IF staining showed that the number of Vimentin+ cells (fibroblasts) significantly increased in GML group, but that the ratio of proliferating fibroblasts (Ki67+, Vimentin+) versus total fibroblasts (Vimentin +) was similar among the four groups (Fig. 5A and 5B), suggesting that the increased fibroblasts aggregation was not due to the change in proliferation. In addition, the effects of MY-1 on the proliferation of fibroblasts were also tested in the primary fibroblasts. CCK-8 assay revealed that MY-1 did not change the proliferation of fibroblasts at a dose of 1–100 nM. At even higher doses of 1 µM and 10 µM, MY-1 suppressed the proliferation of fibroblasts (Fig. 5C). Therefore, this again indicated that the increased number of fibroblasts in the skin wound following MY-1 treatment was not due to the proliferation of the cells. Based on the results from the CCK-8 assay and previous study [25], 10 nM and 100 nM MY-1 were selected as the suitable concentrations for the following studies. In vitro colony forming unit assay and Ki67 staining subsequently confirmed the null effects of MY-1 on fibroblast proliferation. After 7 days of MY-1 incubation, little difference in the number of colony units (Fig. 5D and 5E) and the Ki67+ fibroblast rate was detected (Fig. 5F and 5G), confirming the marginal effect of MY-1 on fibroblast proliferation.

Since MY-1 did not change the proliferation of fibroblasts, one possible explanation for the increased fibroblasts in the wound would be that MY-1 drove fibroblast migration from other sites. To detect whether the MY-1 treatment promotes fibroblast migration, we conducted a cell scratching test on the fibroblast cultures. The results showed that 24 h of MY-1 incubation dramatically enhanced the motility of fibroblasts (Fig. 5H, upper panels; Fig. 5I). That MY-1 enhanced cell motility was further confirmed by the transwell assays, in which MY-1 drove significantly more cells across the 8 μm aperture (especially in 10 nM of MY-1) (Fig. 5H, lowest panel; Fig. 5I). Through wound explant outgrowth, a classic method to evaluate in vitro wound cell migration, MY-1 significantly increased the overall outgrowth of fibroblasts from dermis tissue (Fig. 5J and 5K).

In addition to the increased number of fibroblasts, the enhanced collagen synthesis capacity of fibroblasts (especially the myofibroblasts) might also contribute to the collagen deposition. Therefore, we next evaluated MY-1’s role in collagen synthesis and the myofibroblast differentiation of fibroblast. After treating fibroblasts with MY-1 for 48 h, western blotting was performed to detect the expressions of type I collagen and α-SMA in the fibroblasts. As shown in Supplementary Fig. 6A and 6B, MY-1 did not induce myofibroblast differentiation and collagen synthesis.

Collectively, these findings indicated that the promoted ECM deposition in the GML group was attributed to fibroblast aggregation in the wound area. This effect was primarily due to the enhanced fibroblast migration, rather than the promoted fibroblast proliferation or collagen synthesis capacity (Supplementary Fig. 7).

### PI3K/AKT activation was responsible for MY-1-induced fibroblast migration

**Fig. 6:**
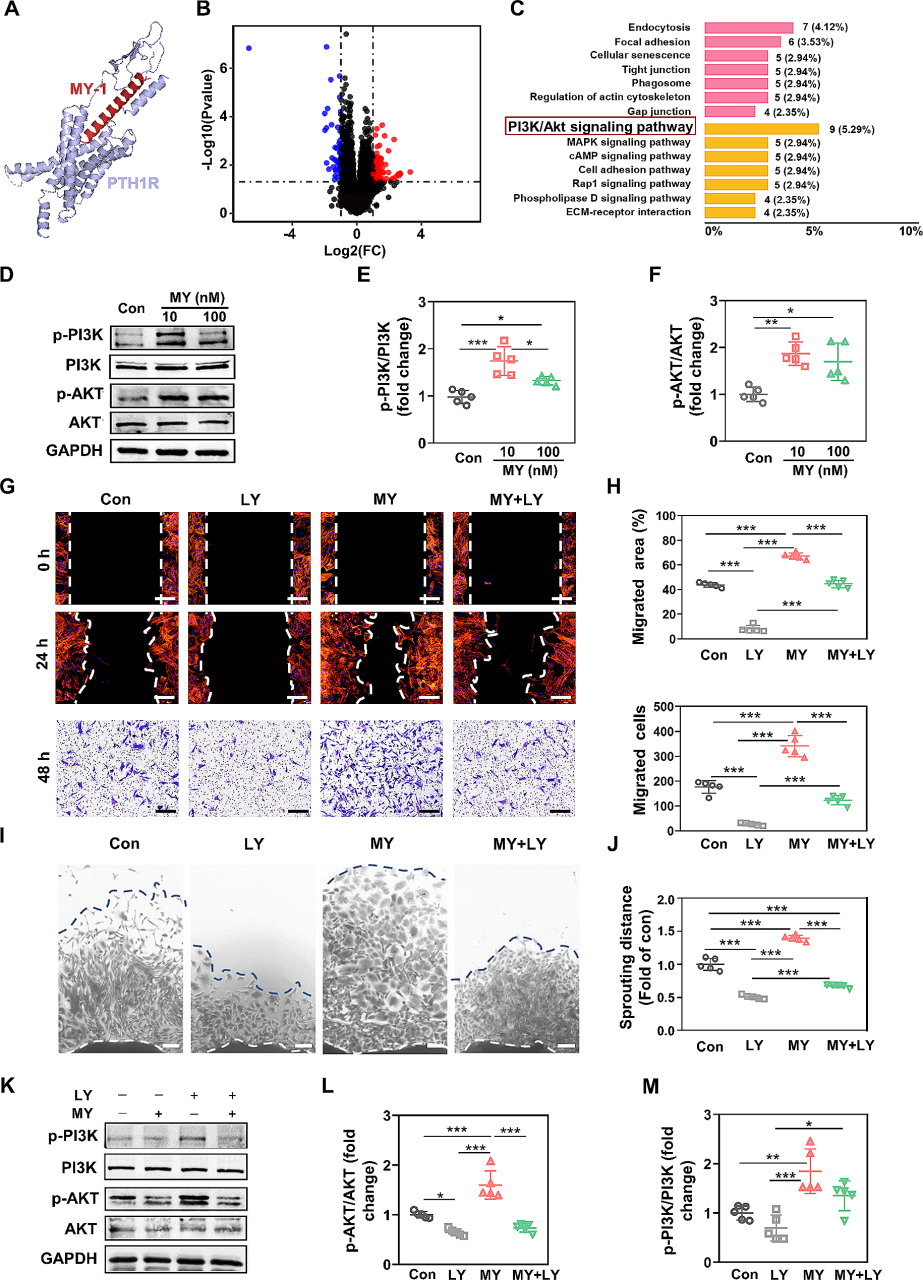
MY-1-enhanced fibroblasts motility was mediated by PI3K/AKT signaling. The docking between MY-1 and PTH1R were simulated by AutoDock software **(A)**. The gene expression profile was explored by transcriptome. Volcano plot images exhibited the gene upregulated or downregulated by MY-1 (**B)**. The signaling pathway that regulated prominently by 10 nM MY-1 was carried out by KEGG pathway analysis **(C)**. The expressions of p-PI3K and p-AKT in fibroblasts were analyzed by western blot **(D)**. The intensity of each band was measure and normalized to total PI3K or AKT then calculated as the ratio of the controls (**E** and **F**). Cell scratching test and transwell assay were employed to investigate the migration of fibroblasts treated with 10 nM MY-1 with or without PI3K inhibitor LY (10 µM) for 24–48 h (**G** and **H)**. Dermis explants assays were performed as described in Fig. 5(**I** and **J**). After the fibroblasts treated with MY-1 with or without 10 µM LY, the expressions of p-PI3K and p-AKT in fibroblasts were quantified as described above (**K**–**M)**. Data were expressed as mean ± SD of triple samples, and three repeated experiments yielded similar results. *, *p* < 0.05; **, *p* < 0.01; ***, *p* < 0.001. Scale bars: 200 μm in (G) and 150 μm in (I)

As described in our previous research, MY-1 is an effective ligand which binds with the PTH type I receptor to initiate the cell migration (Fig. 6A) [25]. To further investigate the downstream mechanisms through which MY-1 improved fibroblast motility, the RNA sequence (RNA-seq) technique was employed, with the results revealing that numerous gene expressions were changed after 3 days of MY-1 treatment (Fig. 6B). Analysis of the Kyoto Encyclopedia of Genes and Genomes (KEGG) revealed that several signaling pathways were activated following the administration of MY-1, in which most numbers of the PI3K/AKT signaling pathway-related genes were changed (Fig. 6C). These results indicated that PI3K/AKT signaling was the most correlated pathway that mediated MY-1-induced fibroblast migration.

We next verified the role of the PI3K/AKT signaling pathway. IHC staining on in vivo samples revealed stronger positive expressions of p-PI3K and p-AKT following the application of GML, thus confirming that PI3K/AKT signaling was involved in the MY-1-mediated wound healing process (Supplementary Fig. 8A–8D). Similarly, the expression of p-PI3K and p-AKT in the MY-1-treated fibroblasts in vitro was significantly increased, while the total PI3K and AKT levels remained consistent among the three groups (Fig. 6D–6F). To confirm the role of the PI3K/AKT pathway in MY-1-treated fibroblast migration, a specific PI3K/AKT pathway inhibitor, LY, was employed. The analysis of cell scratching, transwell assay, and wound explant outgrowth revealed that the enhancement effect of MY-1 on fibroblast motility was significantly inhibited after the addition of LY, confirming the PI3K/AKT pathway’s role in MY-1-enhanced cell migration (Fig. 6G–6J). Moreover, improved protein expression levels of p-PI3K and p-AKT were attenuated by LY (Fig. 6K–6M), which further indicated that the phosphorylation of PI3K and AKT was essential for MY-1’s effect on cell migration.

### Inhibition of PI3K/AKT signaling slowed the fibroblast migration, thus delaying the wound healing and reducing the tensile strength

**Fig. 7:**
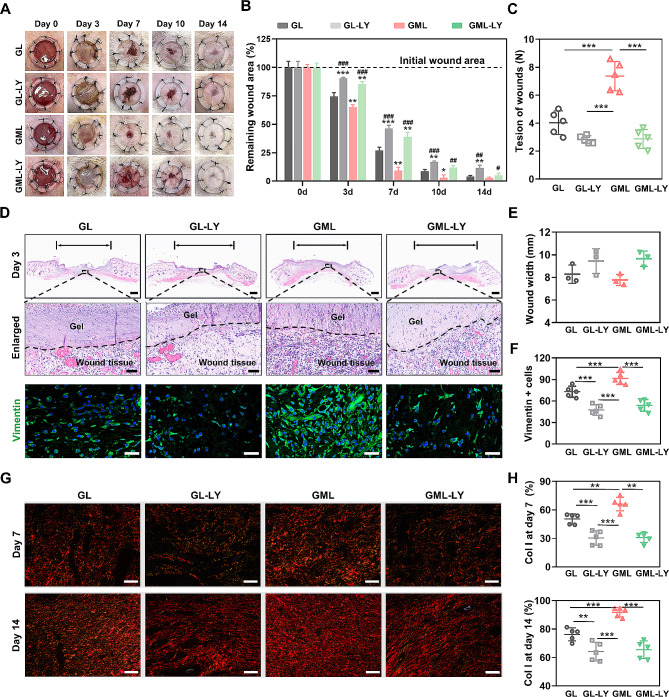
Inhibition of PI3K/AKT signaling attenuated the effect of MY-1 on skin wound healing. Full-thickness skin wound was generated as described in Fig. 4. GL, GL-LY, and GML with or without LY were applied to the defects. Wound closure was investigated at the indicated time point **(A)**. The unclosed wound area was quantified (**B**). The tensile strength of the wounds treated by the above treatments were measured at day 14 (**C**). At day 3, the histological sections of the wound area were prepared and stained with HE and IF of Vimentin **(D)**. The wound width was measured (**E**) and the Vimentin+ cells per 40× field of view were counted (**F**). Picrosirius red staining, and the analyses of type I collagen content (**G** and **H**) were performed as described in Fig. 4. Data were shown as mean ± SD. *, *p* < 0.05; **, *p* < 0.01; ***, *p* < 0.001 versus GL group. #, *p* < 0.05; ##, *p* < 0.01; ###, *p* < 0.001 versus GML group. Scale bars: 3 mm in (A), 1 mm and 50 μm in (D), 200 μm in (G)

To investigate the role of PI3K/AKT signaling in the wound healing process, LY was incorporated into GL or GML and applied at the wound sites, with LY significantly attenuating GML’s effects on wound closure and tensile strength (Fig. 7A–7C, Supplementary Fig. 4D). The wound closure was significantly delayed from day 3 to day 14 (Fig. 7A and 7B, Supplementary Fig. 9A and 9B). The cells’ infiltration into the wound defects induced by MY-1 was also decreased (Fig. 7D, upper panel; Fig. 7E). Moreover, IF staining on Vimentin confirmed that the fibroblast aggregation was significantly inhibited by LY on day 3 (Fig. 7D, lowest panel; Fig. 7F), thus resulting in a notably inhibition of collagen deposition and its maturation at days 7–14 (Fig. 7G and 7H, Supplementary Fig. 9C and 9D). The expression of FN was also decreased by LY (Supplementary Fig. 9E and 9F). The decreased ECM deposition caused by inhibited fibroblast migration in the early stage eventually delayed the wound healing and decreased the tensile strength (Supplementary Fig. 4D). Finally, IHC staining on p-AKT confirmed that the application of LY suppressed the phosphorylate process of AKT, thus blocking the activation of PI3K/AKT signaling (Supplementary Fig. 9G and 9H). Therefore, the PI3K/AKT pathway was identified as playing an indispensable role in mediating MY-1’s effect on fibroblast migration and the skin wound-healing process.

### Rac1 was involved in MY-1-induced fibroblast migration

**Fig. 8:**
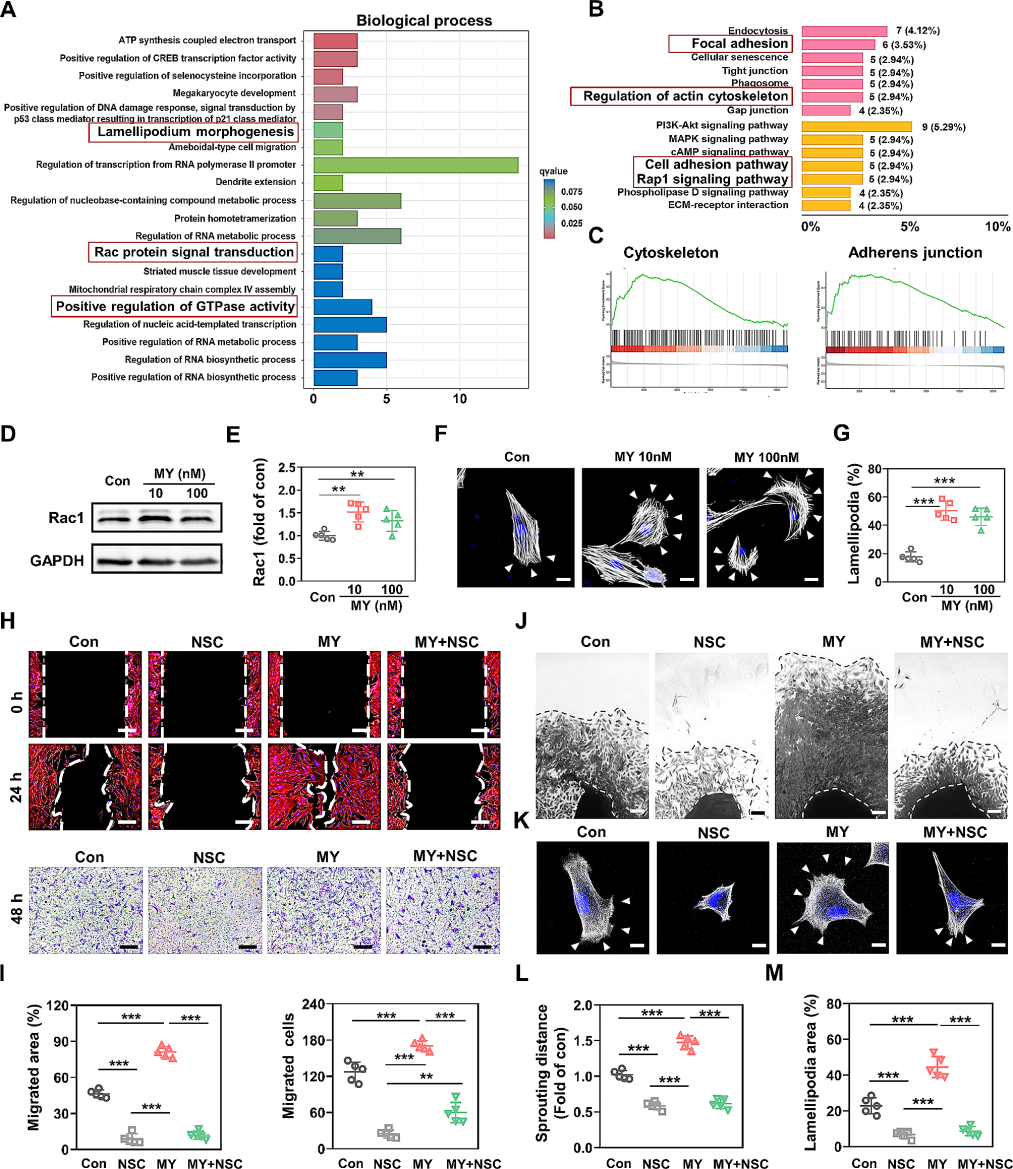
Rac1 was involved in MY-1-induced fibroblast migration. Transcriptome analysis was same as Fig. 6. Gene ontology analysis (**A**), KEGG pathway analysis (**B**), and Gene Set Enrichment Analysis (**C**) were performed to explore the effect of MY-1 on primary dermal fibroblasts. The expression level of Rac1 was detected by western blot (**D**) and the intensity of each band was quantified and normalized to GAPDH and then calculated to the ratio of control group (**E**). The cytoskeleton of migrating cells was stained with rhodamine-phalloidin, lamellipodia area of each cell were measured (**F**) and converted in percentage to the total area of the cell (**G**). Cell scratching test and transwell assays were performed (**H**), the recovered area and migrated cells (**I**) were quantified as described in *materials and methods* section. The cells grew out of dermis explants was investigated and quantified as the farthest distance of cell sprouting (**J** and **L**).. Demonstrations of the cytoskeleton of migrating cells were same as described above (**K** and **M**). Data are expressed as mean ± SD of triple samples and three repeated experiments yielded similar results. *, *p* < 0.05; **, *p* < 0.01; ***, *p* < 0.001. Scale bars: 20 μm in (F) and (K), 200 μm in (H) and 150 μm in (J)

RNA-seq also revealed the genes related to lamellipodia formation, Rac protein transduction, GTPase activity, cytoskeleton, cell adhesion, and cell migration, whereby the cell shape and Rap1 signaling were altered by the treatment of MY-1 (Fig. 8A–8C), indicating MY-1’s effect on cytoskeleton and morphology. Since Rac1 is a key GTPase that is responsible for cell migration, cytoskeleton reconstruction, and lamellipodia formation, we next focused on exploring Rac1’s role. The results demonstrated that the expression level of Rac1 in fibroblasts was improved following MY-1 treatment (Fig. 8D and 8E). Since Rac1-mediated cell migration is closely correlated to lamellipodia formation, we compared the lamellipodia formation capacity among the groups with different treatments by cell scattering assay, with the results showing that after continuous treatment, the MY-1-treated fibroblasts exhibited much wider lamellipodia areas than those in the control group (Fig. 8F and 8G), thus suggesting that MY-1 stimulated the lamellipodia formation. In line with the increased Rac1 expression in cultured fibroblasts, IHC staining in the skin wound samples confirmed these results and more Rac1-positive cell presentation in the GML group (Supplementary Fig. 10A and 10B). Then, a siRNA targeting Rac1 was transfected into the fibroblasts and the repression of Rac1 expression was verified by qPCR and Western blot (Supplementary Fig. 10C–10E). Moreover, after the inhibition of Rac1 expression, MY-1’s effect on cell migration vanished, as demonstrated by the cell scratching and transwell assay (Supplementary Fig. 10F–10I). We next blocked Rac1 activation with a specific Rac1 inhibitor named NSC23766 (NSC). Those fibroblasts co-cultured with MY-1 and NSC also markedly lost their migration capacity in the cell scratching assay, transwell assay (Fig. 8H, lowest panel; Fig. 8I), and wound explant outgrowth (Fig. 8J and 8L). Moreover, NSC inhibited the MY-1-promoted lamellipodia formation (Fig. 8K and 8M). Therefore, the above-mentioned assays confirmed that MY-1-promoted fibroblast migration was closely related to Rac1 signaling.

### Rac1 was regulated by PI3K/AKT signaling

**Fig. 9:**
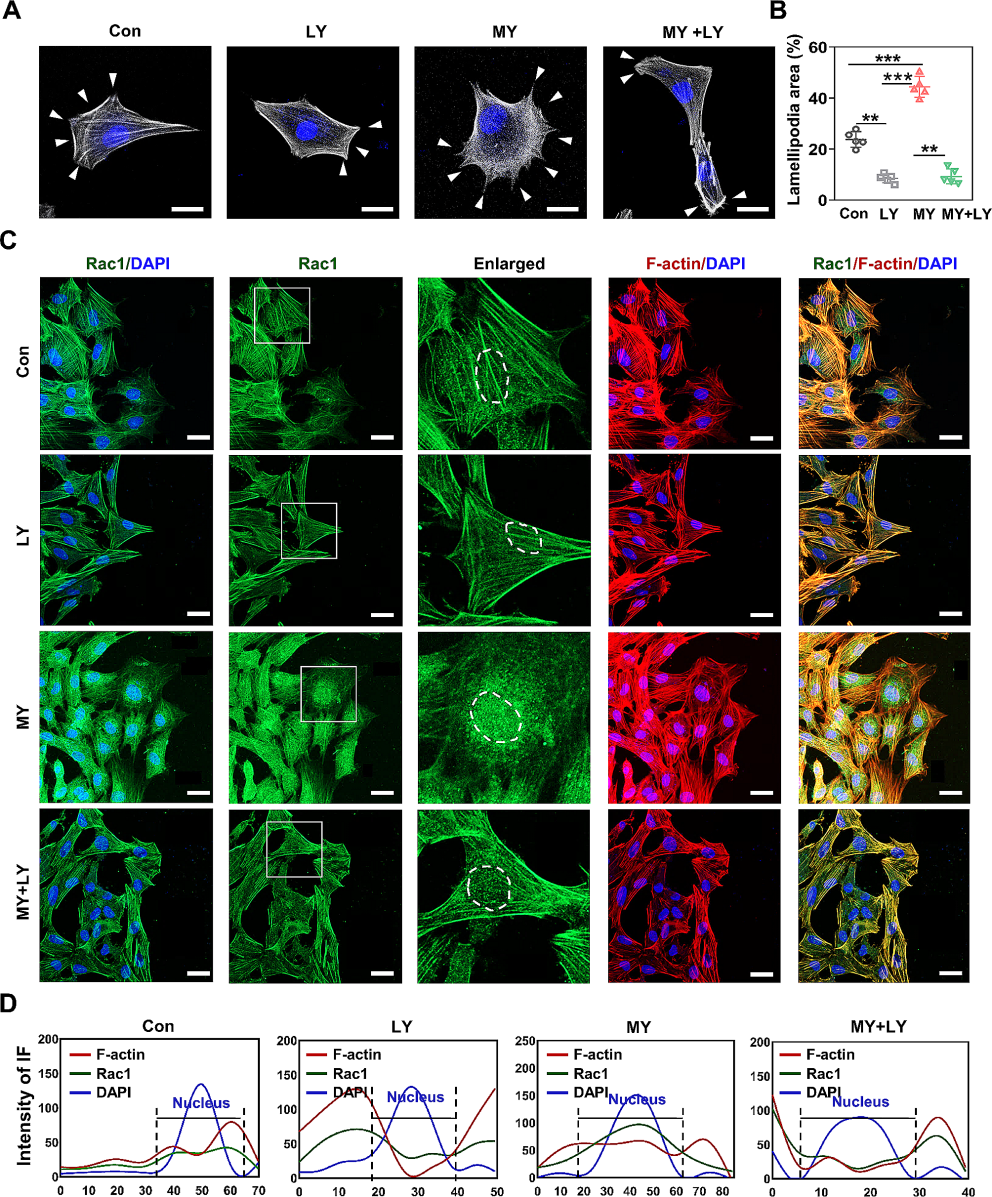
Rac1 expression and its cellular location were regulated by PI3K/AKT signaling. After treated by MY-1, with or without LY (10 µM), lamellipodia area in primary dermal fibroblasts were demonstrated by rhodamine-phalloidin staining (**A**) and quantification (**B**) as described in Fig. 8. IF staining of Rac1 in migrating fibroblasts was performed and the cellular location of Rac1 was revealed by confocal microscopy (**C**). The cellular distribution of Rac1, F-actin and nucleus was measured as described in *materials and methods* (**D**). Data are expressed as mean ± SD of triple samples and three repeated experiments yielded similar results.*, *p* < 0.05; **, *p* < 0.01; ***, *p* < 0.001. Scale bars: 20 μm in (A) and (C)

To investigate whether PI3K regulated Rac1 expression in MY-1-induced fibroblast motility, LY was utilized in the subsequent studies. The results showed that LY decreased MY-1’s positive effect on Rac1 protein expression in vitro (Supplementary Fig. 11A and 11B). In addition, the cell scattering assay revealed that the lamellipodia formation promoted by MY-1 was repressed following LY treatment (Fig. 9A and 9B). To detect the cellular location of Rac1, reported to be closely related to cell migration and invasion [40], the migrating cells (in the scratched area of cell culture) were IF stained to show the Rac1, F-actin, and nuclei. Interestingly, after 12 h of MY-1 treatment, the fascicular arrangement of F-actin became obviously more arranged and located more in the nuclear and perinuclear area (Fig. 9C). Furthermore, this phenomenon was significantly repressed after the addition of LY. Following MY-1 treatment, Rac1 (green line) was more overlapped with nuclear (blue line), and the cellular distribution pattern of F-actin (red line) was closer to that of Rac1 (Fig. 9D). Additionally, the in vivo IHC staining of Rac1 confirmed the in vitro results, based on decreased Rac1-positive cells being detected in the wound areas post-LY administration (Supplementary Fig. 11C and 11D). Collectively, these data demonstrated that MY-1 promotes fibroblast migration through upregulating Rac1 expression and its activation via PI3K/AKT signaling activation.

## Discussion

In the present study, we generated a bioactive material (GelMA–MY@Lipo, or GML) in which MY-1 was slowly released into the skin wound, whereby the wound closure was accelerated and the tensile strength of the wound was enhanced significantly. Further research on fibroblasts and animal models demonstrated that MY-1 promoted fibroblast migration via activating PI3K/AKT signaling and Rac1, which mediated MY-1’s effect on wound healing.

Liposomes are self-assembled nanoscale vesicles capable of encapsulating hydrophobic drugs within their membrane and hydrophilic drugs within the core [32, 41]. Previous studies have demonstrated that encapsulating drugs into the liposomes significantly slowed their release and prevented them from exposure to the harsh external environment (especially the impact of external proteolytic enzymes and pH) [41, 42]. MY-1-loaded liposomes are negatively charged, which could effectively prevent excessive electrostatic interaction between the plasma membrane and negatively charged hydrogel fibers, thus contributing to the prevention of premature drug release [42]. The zeta potential of liposomes was sharply increased after loading with MY-1, which could be attributed to the positive charge that MY-1 carries. Although an increased zeta potential in MY@Lipo might affect their dispersibility and stability, it still maintained certain stability and dispersibility according to the results of TEM, DLS and 3D reconstruction. This phenomenon has also been reported in previous studies where the zeta potential of liposomes increased to 6.42 mV and 12.00 mV, after the encapsulation of VEGF-mimetic or PTH(1–34), respectively, but did not significantly affect their stability and dispersity [32, 38]. Due to the excellent biocompatibility, controllable biodegradation, abundant porous structures, and of course the ideal drug release capacity, GelMA hydrogel was the typical scaffold for hosting cells or drugs [32, 42]. Such a design was reported recently, in which stromal cell derived factor-1α (SDF-1α) was encapsulated in liposomes and then incorporated in GelMA, with the SDF-1α slowly released in vitro [42] that then attracted the migration of mesenchymal stem cells, although the in vivo application has not been reported to date. In our study, after MY-1’s encapsulation in liposomes and incorporation into GelMA, the peptide was released more slowly compared with the unencapsulated MY-1 in GelMA as assayed ex vivo. In the in vivo assay, a slow two-phase releasing pattern was observed. When loaded in GelMA and liposomes, MY–FITC was retained in the wound longer than that loaded in GelMA alone, as per our previous report [25], thus indicating that liposome encapsulation slowed the peptide release, sustained bioactive effects, and was undoubtedly beneficial for deep tissue regeneration. This phenomenon might be attributed to hydrogen bonding between liposomes and GelMA, as well as the two-stage release pattern (MY@Lipo’s release from the degenerating hydrogel and encapsulated MY-1’s slow release from MY@Lipo) after MY@Lipo was incorporated into GML, as demonstrated in earlier research [42]. Collectively, GML hydrogel achieved a controlled release of MY-1 and effectively maintained the biological activity of this short peptide for a longer period of time, which had a positive effect on wound repair and tensile strength.

According to the World Union of Wound Healing Societies, and the SWD Grading System developed by the Core Expert Working Group, SWD was classified into four grades (I–IV) based on the separated tissue layers of skin, subcutaneous, muscle, and deep fascia [39], whereby skin wounds generated in rats could mimic the Grade III SWD to some extent. By utilizing such an animal model, the GML system obtained the ability to enhance the pace of wound closure and increased ECM formation, as well as increasing the tensile strength when measured at day 14. Interestingly, more fibroblasts appeared in the wound area 3 days post-GML application, followed by a quicker deposition and improved assembly of type I collagen at a later time. Increased fibroblasts were not caused by proliferation; the most likely explanation is that MY-1 drove fibroblasts from the adjacent area. MY-1’s positive effect on cell migration was confirmed by in vitro studies of primary dermal fibroblasts. Both the cell scratching test and transwell assay identified that MY-1 promoted cell migration. Similar results were obtained in other experiments where the migrations of bone marrow stromal cells [36], vascular endothelial cells, and dental pulp stem cells were also promoted by MY-1 (data not shown). As more fibroblasts reached the skin defect, more mature ECM was secreted, as shown by the increased amount and superior assembly of type I collagen in the wounds treated by GML. This conclusion was further validated in the subsequent in vivo studies. Through inhibiting fibroblast aggregation in the wound with a PI3K inhibitor at an early stage, ECM secretion and tissue tension in the GML-treated wounds significantly decreased at day 7–14. Due to MY-1 promoting collagen deposition in wounds, one concern was that MY-1 might lead to scar formation. Therefore, we evaluated the scar formation in the wound area after 14 days of GML treatment and found that the GML group did not exhibit larger scar areas. In HE staining, a significant regeneration of hair follicles was observed in the GML group, which contrasted with the lack of skin appendages typically seen in scar tissue. Moreover, Masson’ trichrome staining revealed that the arrangement of newly formed collagen in the GML group was more similar to normal skin than scar tissue. Thus, we concluded that the topical application of MY-1 did not promote scar formation.

Unlike PTH(1–34) and its analogs, which promoted tissue repair because of their multifunctional activities including, but not limited to promoting cells proliferation, inducing their differentiation, and accelerating fibroblast migration [29, 43], MY-1 is special due to its ability to concentrate on cell migration. The rationale of such differences may be caused by selective signaling pathways initiated by MY-1. Based on the bone metabolism literature, a variety of important signaling mediators such as phospholipase C, protein kinase C (PKC), protein kinase A, and β-arrestin were activated after PTH(1–34) stimulation, with signaling pathways including Wnt/β-catenin and TGF-β/Smads then initiated. Through multiple signaling pathways, and other sophisticated mechanisms, a broad range of cells such as BMSCs and osteoblasts were affected and osteogenic differentiation and bone formation were accelerated. Nevertheless, the key signaling pathways that mediate PTH’s impact on fibroblasts remain unclear. *Yao et al.* reported that the TGF-β/Smad3/mTOR pathway was involved in the effect of the systemic administration of PTH on acute skin wound healing [29]. Shen et al. pointed out that β-catenin and the Akt/Erk1/2 pathway were involved in PTHrP2’s effect on the skin wound healing process to increase the regulation of fibroblast migration and differentiation [43]. Since MY-1 selectively activated PKC and β-catenin signaling, its role in fibroblast migration might be mediated by different pathways, as compared with PTH [44]. On the other hand, to achieve positive effects, PTH(1–34) has be released in a precise manner, or a negative effect might occur; therefore, MY-1 is superior to PTH(1–34).

In order to ensure that the signaling pathway has the maximal influence, transcriptomics was employed and PI3K/AKT signaling was subsequently verified as the most relative pathway. The results of high throughput screening were verified by in vitro and in vivo experiments. Hence, we demonstrated that MY-1-induced fibroblast migration was dependent on the activation of the PI3K/AKT pathway. To detect the downstream targets regulated by MY-1-activated PI3K/AKT signaling, we further analyzed the data of RNA-seq and Rac1 was resolved. In our experiments, the expression of Rac1 was significantly upregulated in the wound areas and cultured fibroblasts following MY-1 administration, suggesting that Rac1 was the downstream target of MY-1. After repressing Rac1 expression with siRNA or its specific inhibitor, NSC, the migration of fibroblasts upon MY-1 treatment was significantly reduced. As a member of the Rho family of GTPases, Rac1 is a well-known pleiotropic regulator of multiple cellular processes, especially in regulating cytoskeleton dynamics, cell–cell junction, and cell motility [40, 45]. In migrating cells, GTP-bound Rac1 mediates the actin polymerization to form lamellipodial protrusions at the leading edge through activation of the PAK and WAVE complex, thereby providing the driving force of cell movements [46, 47], with such a phenomenon also presenting in our experiments. It was also reported that Rac1 accumulation in the nucleus could change the Rac1–RhoA ratio in the cytoplasm, which subsequently increased RhoA signaling in the cytoplasm and promoted a highly invasive phenotype [48]. In skin wound healing, the positive role of Rac1 is well described in previous studies, whereby Castilho et al. reported that the deletion or inhibition of the Rac1 gene in the epidermis led to delayed skin wound healing [49] while Tang et al. demonstrated that Rac1 was upregulated in wound areas, and that the inhibition of Rac1 using microRNA-00b/c-3p resulted in a delayed wound healing process [50].

The relationship of upregulated Rac1 and PI3K/AKT signaling activation was then clarified. After the administration of LY, the expression of Rac1 was significantly repressed, and accompanied by reduced cell migration capacity. Moreover, the nucleus accumulation of Rac1, a symbol of the improved migration capacity of MY-1-induced fibroblasts, was remarkably repressed following the administration of LY. PI3K/AKT-Rac1 is an important pathway involved in fibroblast migration and wound healing. bFGF was reported as utilizing such a pathway to protect the blood–brain barrier [51] and promote wound healing [52, 53] by increasing the level of GTP-bound Rac1. Our results indicated that Rac1 translocation into or close to nuclei was a relevant sign of its activation following MY-1 treatment.

To conclude, the present study revealed that GML accelerated wound closure and increased tensile strength by enhancing fibroblast migration, which led to increased fibroblast aggregation and subsequent collagen deposition in the wound area. This study also revealed that the MY-1-enhanced fibroblast migration capacity is primarily related to the PI3K/AKT signaling and the subsequent activation of its downstream targets, the Rac1 signaling. These findings indicated that the GelMA-controlled local release of liposome encapsulated MY-1 could present an ideal wound healing agent with the capacity to prevent WD or SWD, and thus more systemic investigations need to be conducted in the future.

### Electronic supplementary material

Below is the link to the electronic supplementary material.

Supplementary Material 1

## Data Availability

No datasets were generated or analysed during the current study.
