# Supplementary material for: Injective hydrogel loaded with liposomes-encapsulated MY-1 promotes wound healing and increases tensile strength by accelerating fibroblast migration via the PI3K/AKT-Rac1 signaling pathway
Source: J Nanobiotechnology. 2024 Jul 5;22:396. doi: 10.1186/s12951-024-02666-3 (PMC11225333; doi:10.1186/s12951-024-02666-3)
Supplement: Supplementary file 1 — Supplementary Material 1 [file 12951_2024_2666_MOESM1_ESM.docx]

**Supplementary data**

*of*

**Injective hydrogel loaded with liposomes-encapsulated MY-1 promotes wound healing and increases** **tensile strength by accelerating fibroblast migration via the PI3K/AKT-Rac1 signaling pathway**

Chunhao Zhou^1#^, Zhihai Cai^1^^#^, Jialiang Guo^1#^, Chengfu Li^1^, Chenghe Qin^2^, Juanwen Yan^3*^, Dehong Yang^1*^

^1^ Department of Orthopedics - Spinal Surgery, Nanfang Hospital, Southern Medical University, Guangzhou 510515, China

^2^ Department of Orthopedics - Traumatology, Nanfang Hospital, Southern Medical University, Guangzhou 510515, China

^3^ Department of Stomatology, Nanfang Hospital, Southern Medical University, Guangzhou 510515, China

**^*^ Joint corresponding authors:**

Dehong Yang

Department of Orthopedics - Spinal Surgery

Nanfang Hospital, Southern Medical University

1838 Guangzhou North Avenue

Guangzhou510515, China

Tel: (+86) 186 2008 7638

Fax: (+86) 206 278 7195

Email: [942653397@qq.com](mailto:942653397@qq.com)

**^#^ The co-first authors:** Chunhao Zhou, Zhihai Cai and Jialiang Guo contributed equally to this work.

**Running title**: Wound healing and hPTH(3-34/29-34)

**Keywords:** Wound healing, Skin, PI3K/AKT, Rac1, Cell migration, hPTH(3-34/29-34)

MY-1 was patented in China (No. 201610765250.3), and an international patent application (PCT/CN2018/114335) has been submitted.


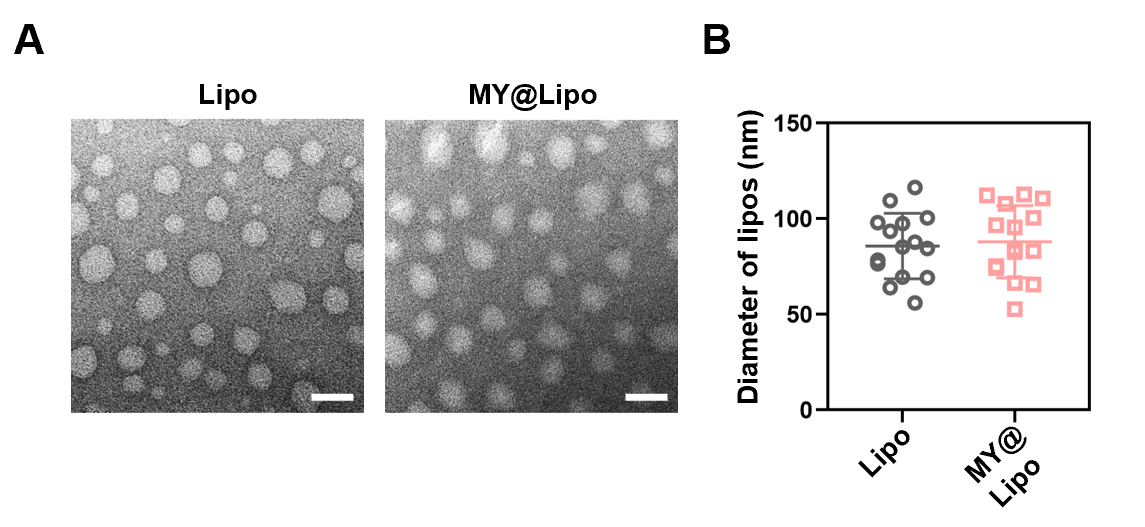


**sFigure 1. TEM images of empty liposomes and MY@Lipo immersed in water for 7days.** Empty liposomes and MY@Lipo were immersed in water for 7 days and the diameters of liposmoes evaluated by TEM(A). The diameters of two groups were measured (B). Scale bars: 100 μm in (A).


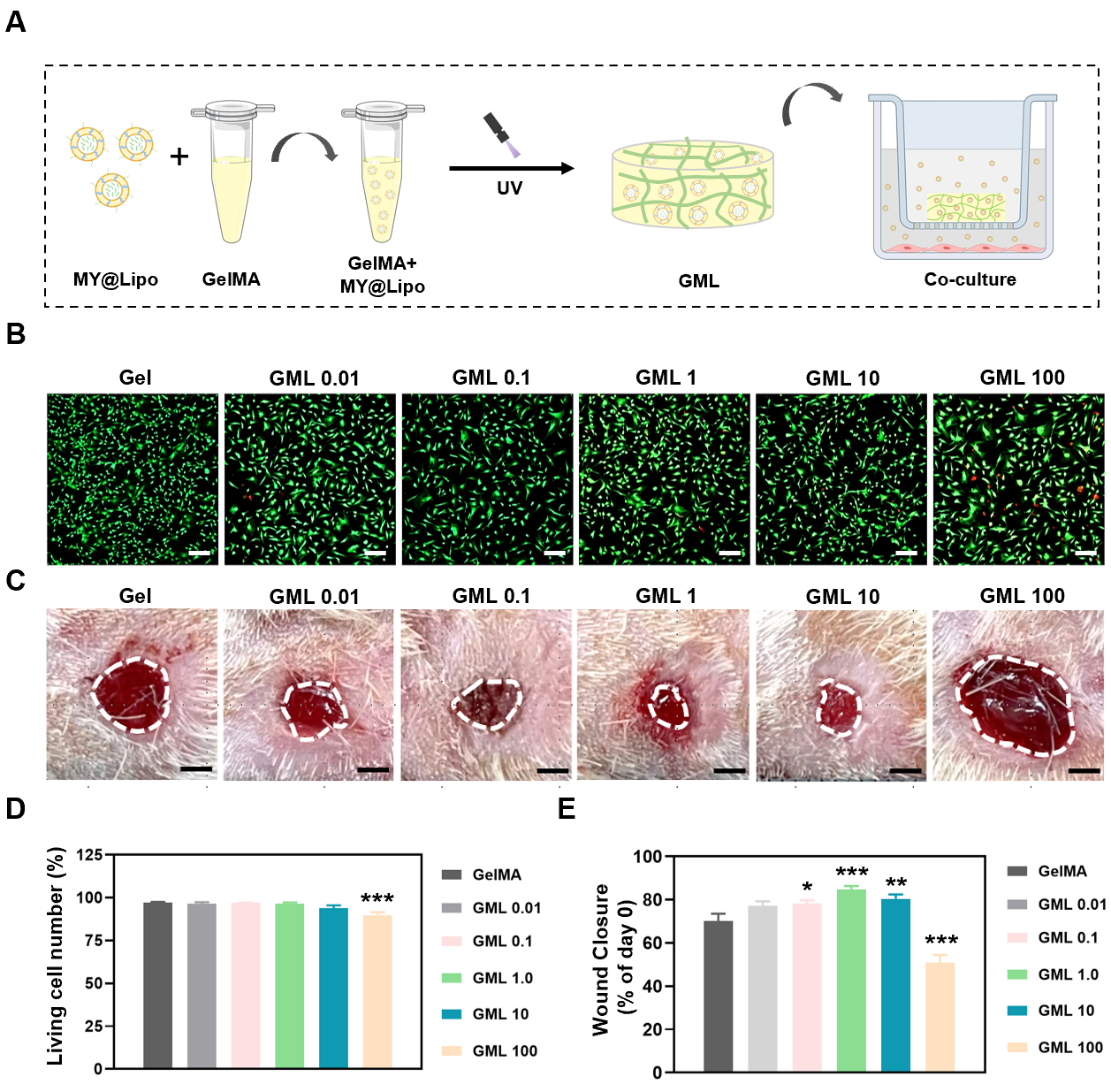


**sFigure 2. The effects of releasing system composed with GelMA hydrogel and lipsomes containing MY-1 peptide accelerated skin wound healing in rat models in a dose response manner.** Diagram of the cell culture systems. The GelMA containing the liposomes encapsulated with different amount of MY-1 peptide was placed in the upper chamber of an 8 µm pore-size transwell plate and the primary dermal fibroblasts were cultured in the lower chamber **(A)**. After 24 h, the cells were stained using a Calcein-AM/PI kit. Living cells exhibited green fluorescence and dead cells were stained in red **(B)**. Full thickness skin defect rat model with a diameter of 15 mm was generated as described in *materials and methods*. GelMA loaded with serial doses of MY-1 were filled into the skin defects. After 7 days, wound closure was investigated **(C)**. The living cell percentage under different concentration of MY-1 peptide was quantified **(D)**. The wound closure rate under different concentration of MY-1 peptide (**E**). Data are expressed as mean±SD. *, p<0.05; **, p<0.01; ***, p<0.001 vs GelMA. Scale bars: 20 μm in (B), 3 mm in (C).


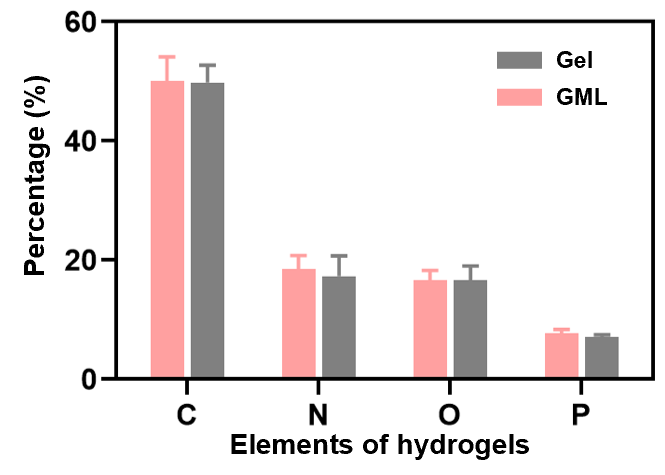


**sFigure 3. The Chemical elements of carbon (C), nitrogen (N), oxygen (O) and phosphorus (P) in Gel and GML analyzed by EDS spectrum.**


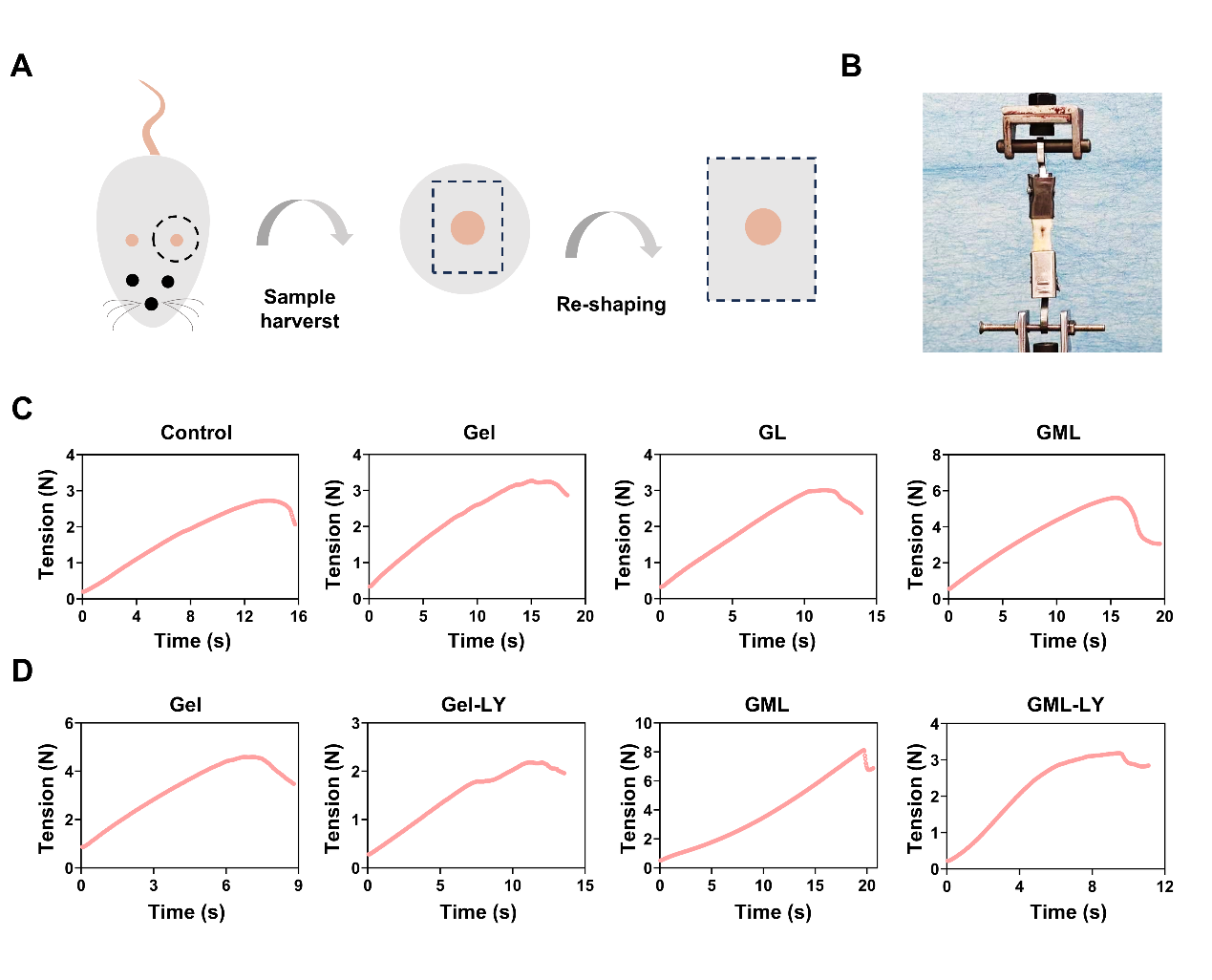
**sFigure 4. Tensile strength measurement.** Rat skin wound model generation and treatments were described in Figure 2. At day 14, centered on the wound, fresh full-thickness rectangular skin samples sized 1 cm × 0.5 cm were isolated (**A**). Then the samples were fixed on tensiometer of the electronic universal testing machine (**B**). The highest tension before the sudden decrease (breakage of the sample) was defined as tensile strength of each sample (**C**). Rat skin wound model generation and treatments were described in Figure 7. Fresh full-thickness rectangular skin samples were isolated at day 14. The samples were subjected to tensile strength measurement. The highest power before the sudden decrease was defined as tensile strength of each sample (**D**).


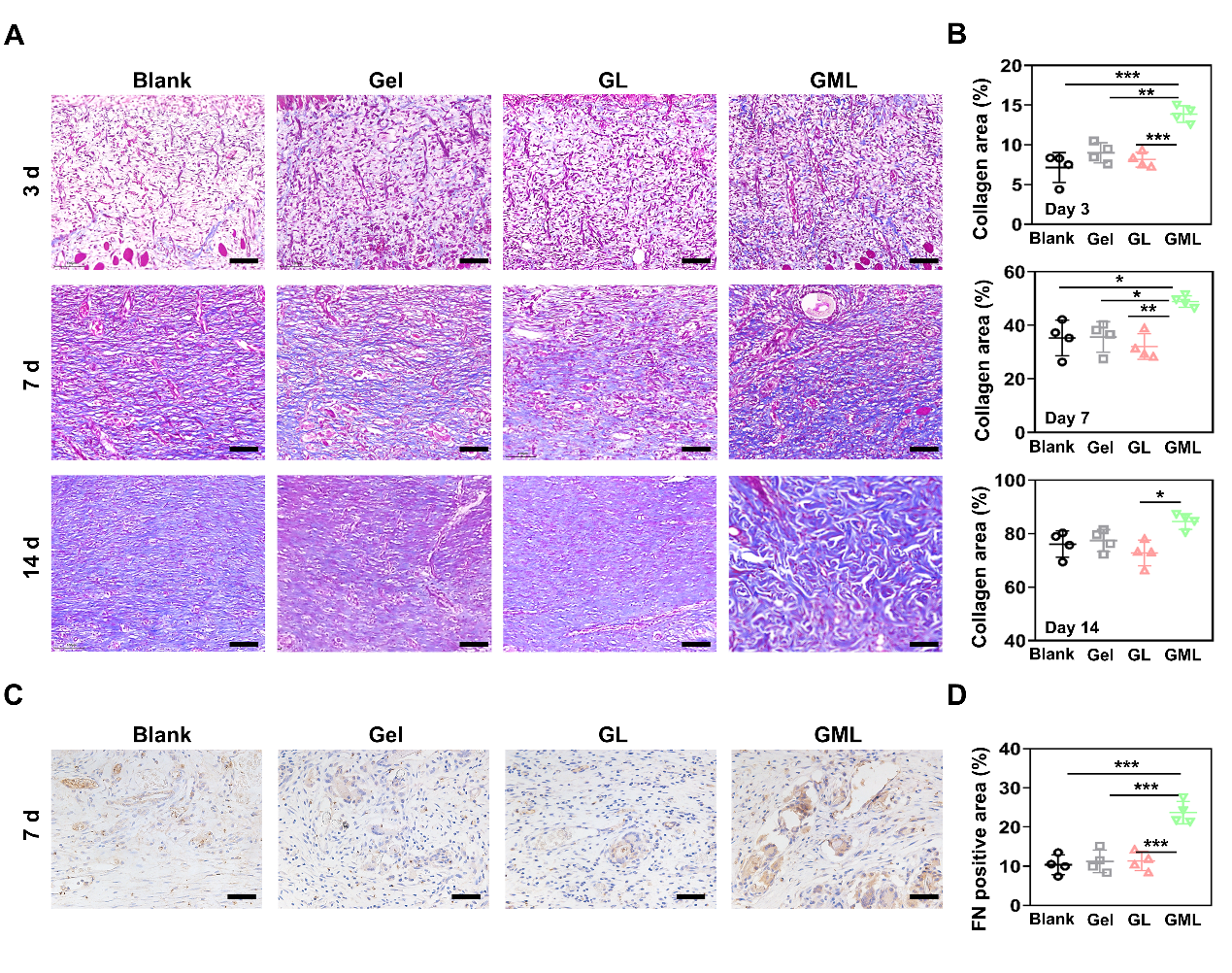
**sFigure 5. GML increased ECM deposition in wound healing.** Skin defect generation and application of GelMA and MY-1 were as a description in Figure 3. Seven or 14 days after hydrogel and peptide application, collagen deposition was displayed by Masson’s trichrome staining (**A**) and quantified as the percentage of collagen area (blue) to total tissue area (**B**). IHC staining of FN in wound was performed at day 7 (**C**), and the intensity of FN (brown black) was quantified and normalized to that of blank (**D**). Data are expressed as mean±SD of 6 animals. *, p<0.05; **, p<0.01; ***, p<0.001. In statistical charts, the groups of blank, Gel, GL and GML were indicated as black circle, gray square, red and green triangle respectively. Scale bars: 200 μm in (A) and (C).


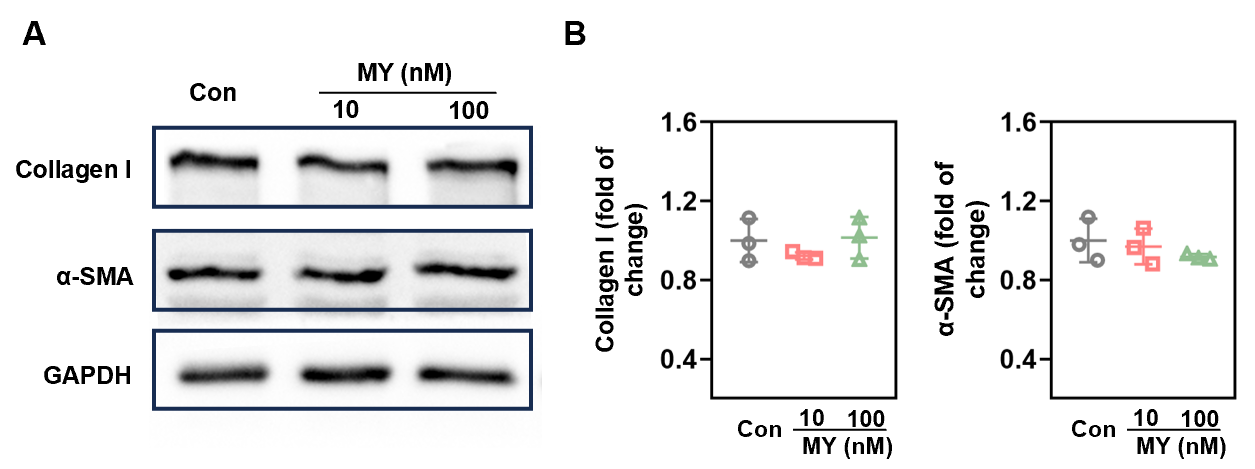


**sFigure 6. MY-1 did not induce type I collagen secretion and differentiation of myofibroblasts.** fibroblasts were treated with/without MY-1 for 48 h and the expressions of Collagen I and α-SMA were measured using western blot (A). The intensity of each band was measure and normalized to GAPDH then calculated as the ratio of the controls (B).


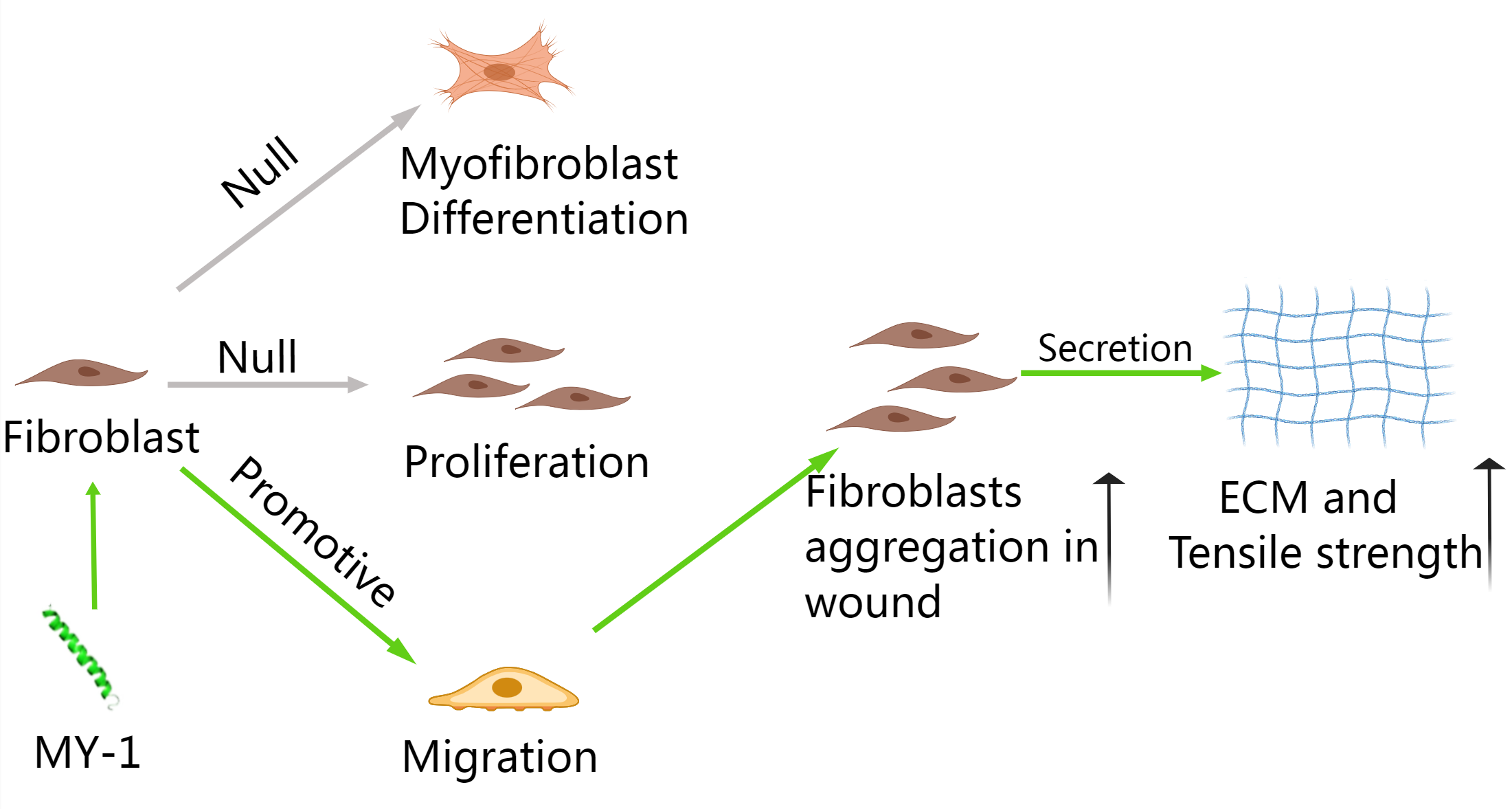


**sFigure 7. The diagram of how MY-1 promotes wound healing and tensile strength by accelerating fibroblast migration.**


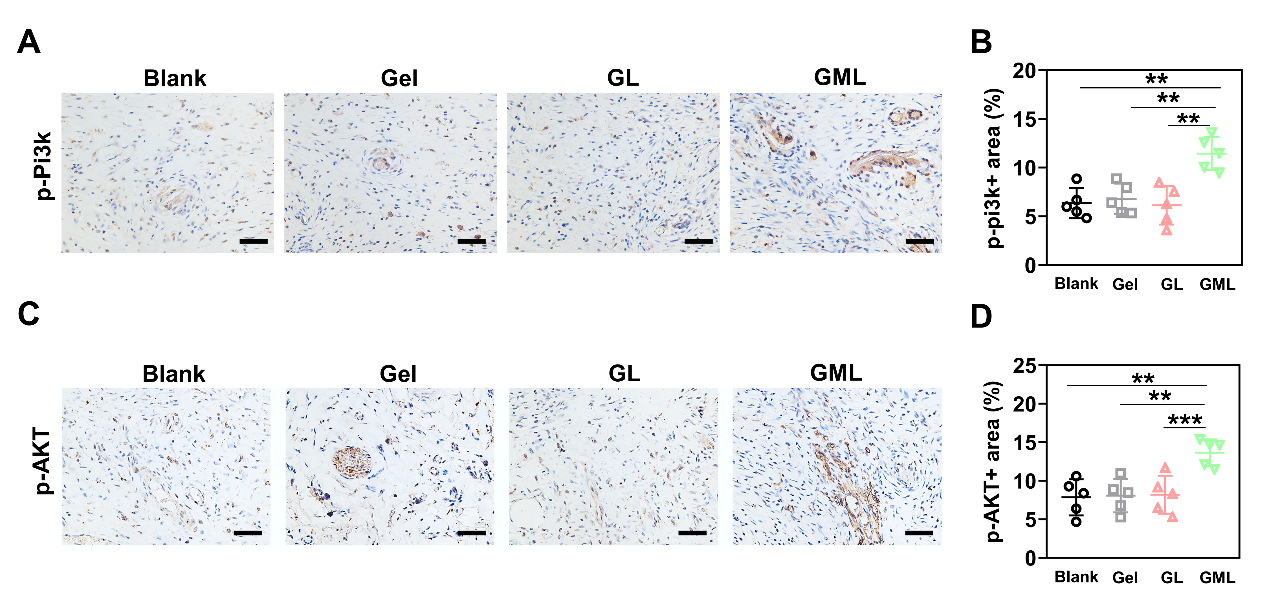


**sFigure 8. GML increased on skin wound regeneration and ECM deposition.** Skin defect generation and application of PBS, Gel, GL and GML were performed as a description in Figure 4. IF staining of p-PI3K and p-AKT was performed in skin wound sections (**A** and **C**). Scale bars: 200 μm in (A) and (C).


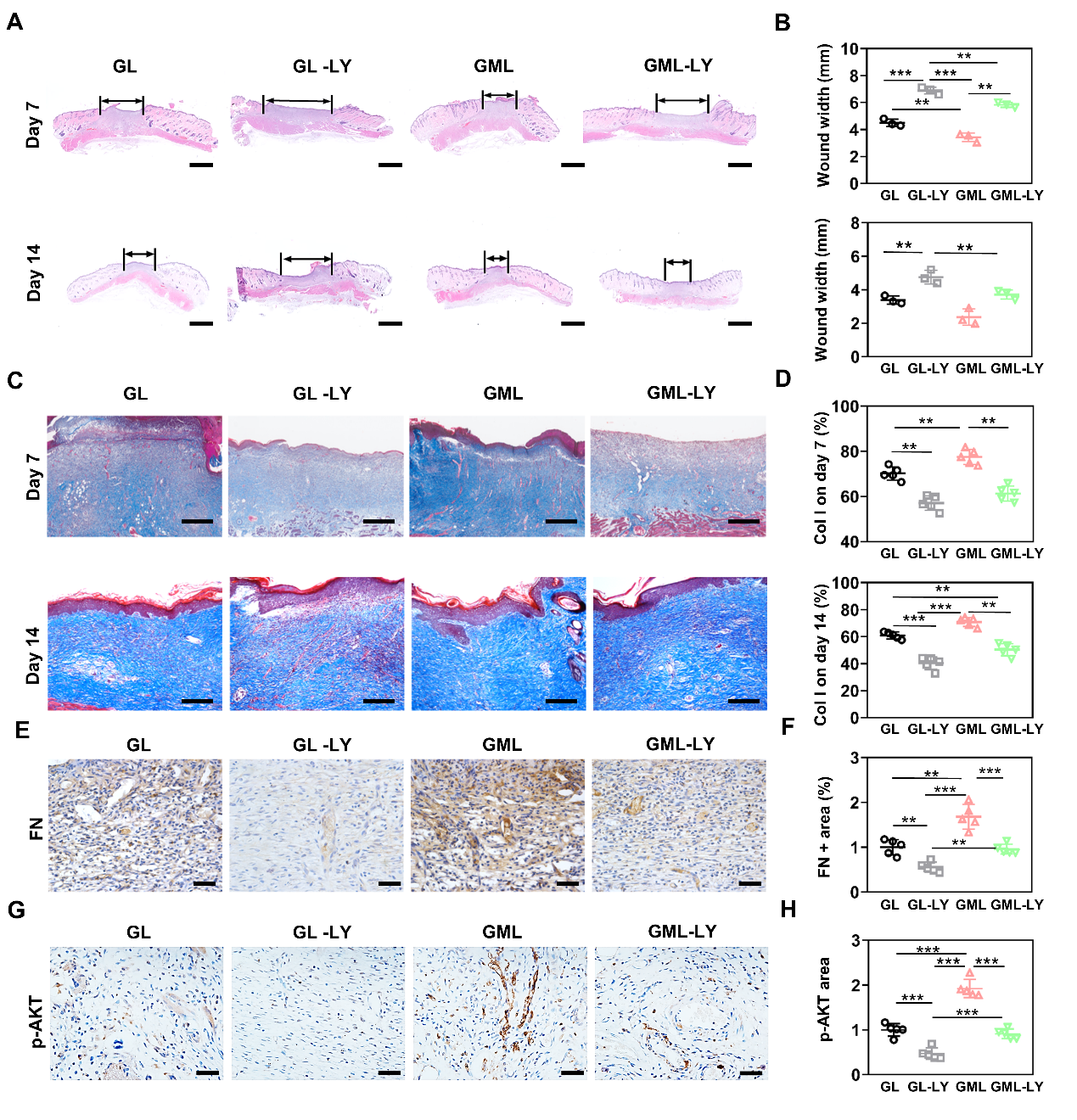


**sFigure 9. PI3K/AKT signaling mediated the effect of MY-1 on skin wound regeneration and extracellular matrix deposition.** Skin defect generation and application of GelMA loaded with Lipo and MY@Lipo as well as LY were performed as a description in Figure 6. Histological images of day 3 were shown in Figure 6. Histological images of day 7 and day 14 were shown in **A** and **B**. Collagen deposition at day 7 and day 14 was stained by Masson’s trichrome (**C**) and quantified by calculating the percentage of blue area over total tissue area (**D**). The effect of LY on the expression of FN and p-AKT was displayed by IHC staining (**E** and **G**) and the quantification of FN and p-AKT was performed as described above (**F** and **H**). Data are expressed as mean±SD of 6 animals. *, p<0.05; **, p<0.01; ***, p<0.001. Scale bars: 250 μm in (A), 200 μm in (C), (E) and (G).


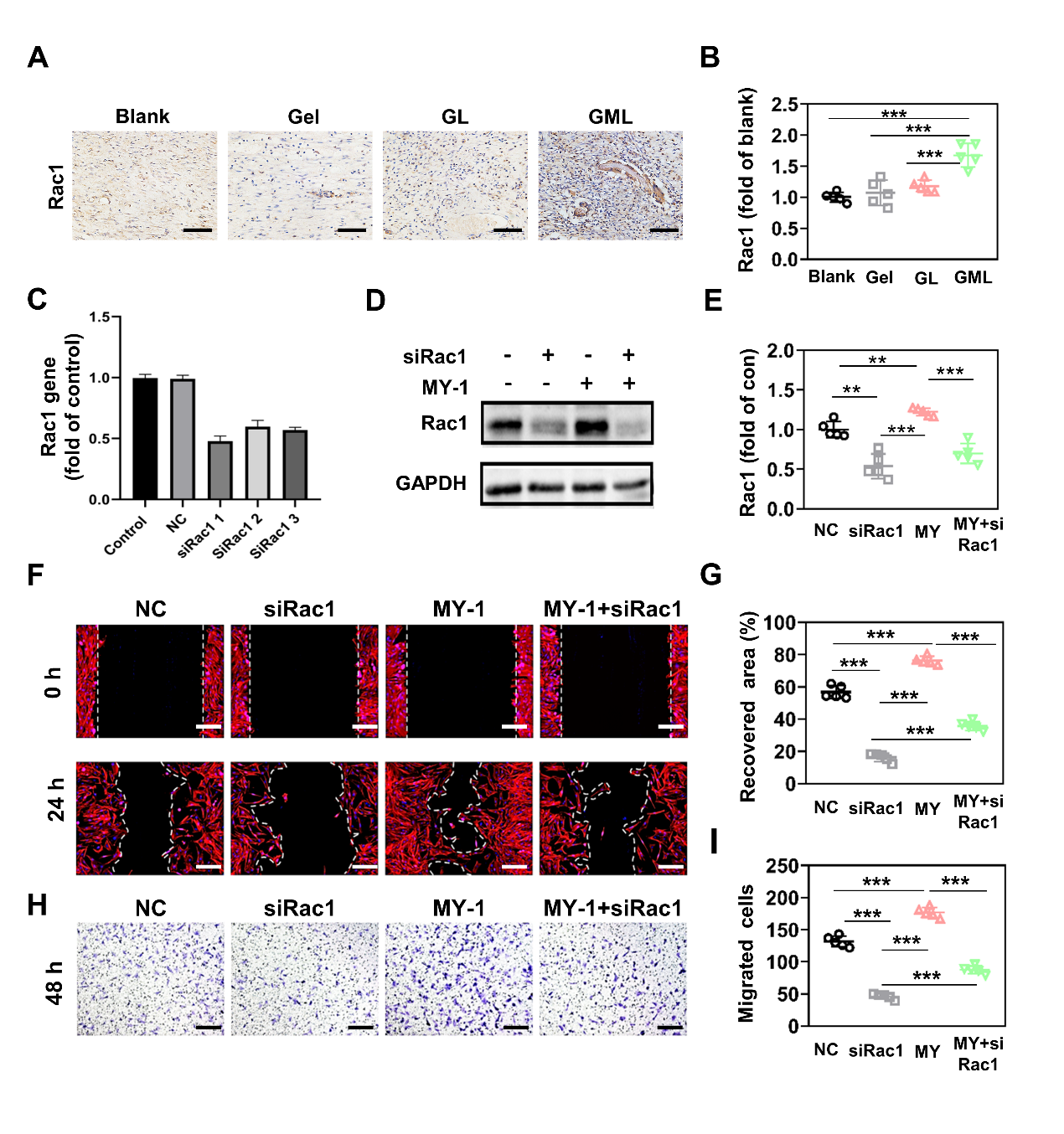
**sFigure 10. Rac1 signaling mediated the effect of MY-1 on fibroblast migration and wound healing.** Skin defect generation and treatment were as a description in Figure 4. IHC staining of Rac1 was performed (**A**) and quantification of the expression was performed and normalized to blank (set at 1.0) (**B**). siRNA technique was employed to blunt the expression of Rac1. The function of Rac1 siRNA (siRac1) on gene and protein expression of Rac1 was verified by realtime PCR (**C**) and western blot (**D and E**). Cell scratching test and transwell assay were employed to detect siRac1’s effect on fibroblast migration induced by MY-1 (**F to** **I**). Data are expressed as mean±SD of 6 animals. *, p<0.05; **, p<0.01; ***, p<0.001. Scale bars: 200 μm in (A), 100 μm in (F) and (H).


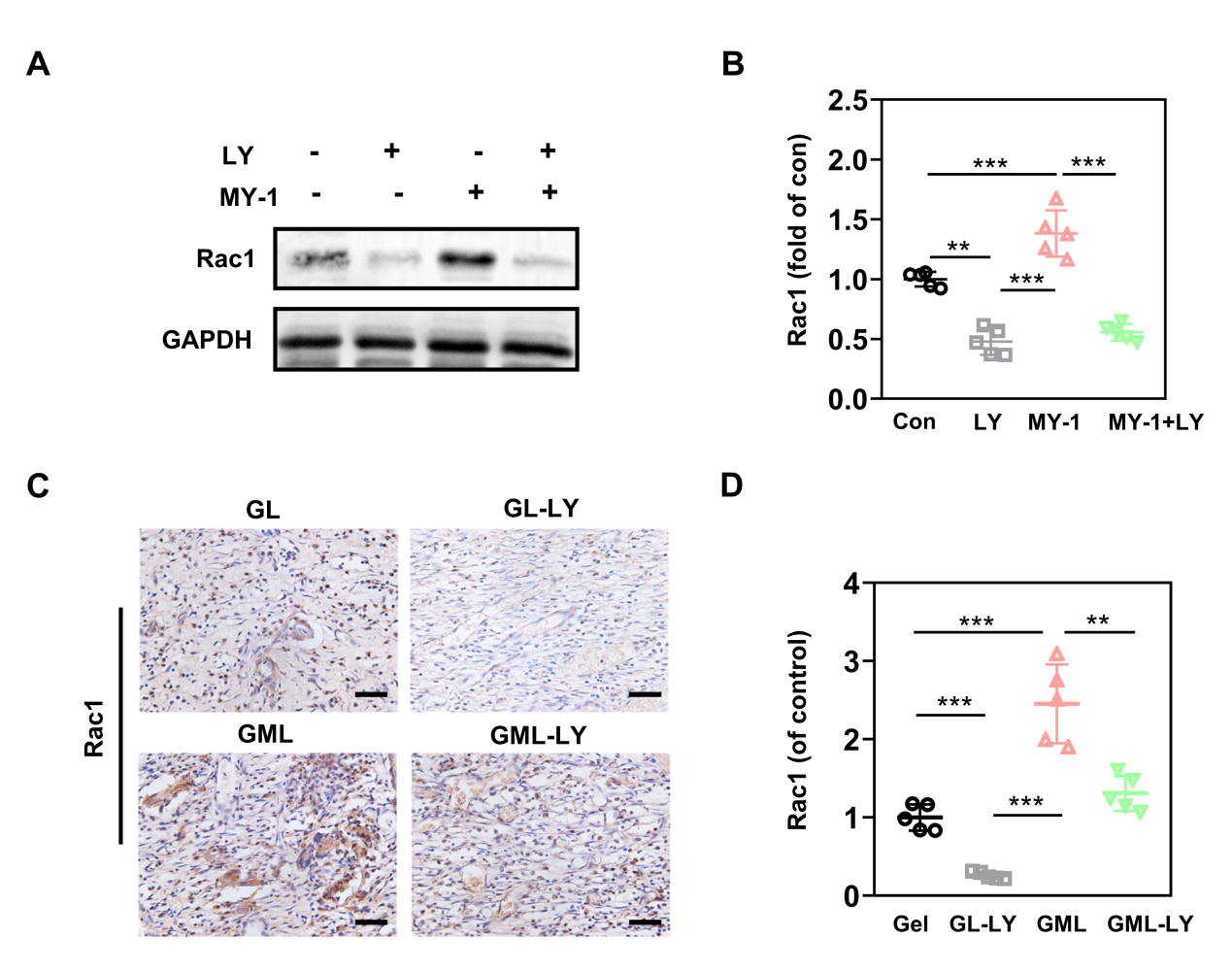


**sFigure 11. The effect of MY-1 on expression of Rac1 was depended on PI3K/Akt signaling.** Primary rat dermal fibroblasts were treated by PBS, 10 μM LY, 10 nM MY-1 or MY-1+LY and the cell lysate was extracted and applied for western blot analysis (**A**). The expression of Rac1 was qualified and normalized to control (set at 1.0) (**B**). The histological sections isolated at day 7 as described in Figure 6 were subjected to IHC to demonstrate Rac1 expression (**C**). The expression level of Rac1 was quantified as described in *materials and methods* (**D**). Data are expressed as mean±SD of 6 animals. *, p<0.05; **, p<0.01; ***, p<0.001. Scale bars: 200 μm in (C).
